# The Epidemiological and Toxicological Intersection of Air Pollution and Dementia

**DOI:** 10.1007/s44169-025-00092-6

**Published:** 2025-10-10

**Authors:** Sumasri V. Kotha, Grace Kuo, Sachin V. Kammula, Liuhua Shi, Xuan Zhang, Pengfei Liu, Xiaobo Mao

**Affiliations:** 1 Neuroregeneration and Stem Cell Programs, Institute for Cell Engineering, Johns Hopkins University School of Medicine, Baltimore, MD 21205 USA; 2 Department of Neurology, Johns Hopkins University School of Medicine, Baltimore, MD 21205 USA; 3 Vertex Pharmaceuticals, Boston, MA 02210 USA; 4 Department of Life and Environmental Sciences, University of California, Merced, CA 95343 USA; 5 School of Earth and Atmospheric Sciences, Georgia Institute of Technology, Atlanta, GA 30332 USA; 6 Adrienne Helis Malvin Medical Research Foundation, New Orleans, LA 70130-2685 USA; 7 Institute for NanoBioTechnology, Johns Hopkins University, Baltimore, MD USA; 8 Department of Materials Science and Engineering, Johns Hopkins University, Baltimore, MD USA

## Abstract

Accumulating evidence indicates that exposure to air pollution can be strongly associated with the development of dementia. However, different components and species of air pollution have been found to have varying associations with incidence of dementia in exposed populations. Among the most broadly studied pollutants in this context are particulate matter (PM), nitrogen dioxide (NO_2_), ozone (O_3_), and sulfur dioxide (SO_2_). Here, we offer a joint toxicological and epidemiological review of the association of each of these well-studied pollutants with dementia, as well as other less-studied pollutants, such as black carbon (BC) and metals (both independently potent components of PM), carbon monoxide (CO), and polyaromatic hydrocarbons (PAHs). Sources and properties of these pollutants are considered in tandem with studies regarding their epidemiological association with dementia and the possible biological basis of these associations. By considering this joint perspective, we identify the pollutants with the greatest association with dementia incidence. Further, we offer a classification of pathological mechanisms and subtypes of dementia associated with exposure to the aforementioned pollutants. Despite variance in exposure assessment, dementia diagnosis and follow-up, and source population, our qualitative review indicates exposure to air pollutants, especially PM_2.5_ and NO_2_, is associated with increased risk and incidence of dementia. Increasing evidence shows exposure to specific pollutants is tied to pathological mechanisms and dementia subtypes, as summarized here.

**Supplementary Information:**

The online version contains supplementary material available at 10.1007/s44169-025-00092-6.

## Introduction

Dementia encompasses a spectrum of neurological disorders characterized by progressive cognitive decline resulting from neuronal loss in the brain. Subtypes of dementia, including Alzheimer’s Disease (AD), Lewy Body Dementia (LBD), vascular dementia (VaD), and frontotemporal dementia (FTD), differ in their underlying pathologies, symptoms, and progression patterns. For instance, AD is marked by amyloid-beta (Aβ) plaques and hyperphosphorylated tau co-pathology, while LBD involves alpha-synuclein (α-syn) aggregation presenting more pronounced cognitive impairments than patients with Parkinson’s disease (PD). VaD results from disrupted blood flow to the brain, while FTD is associated with tau or TDP-43 pathology. Despite these distinctions, dementia subtypes share overlapping features, complicating diagnosis and treatment. Identifying specific links between air pollutants and dementia subtypes offers a unique opportunity to uncover molecular mechanisms and guide targeted interventions (Akhtar et al. 2024).

Recent studies suggest that air pollution may play a significant role in the onset and progression of dementia. Inhaled pollutants such as particulate matter (PM), nitrogen dioxide (NO₂), and ozone (O₃) are known to induce oxidative stress and neuroinflammation, initiating destructive biological processes that accelerate neuronal damage (Wilker et al. 2023). Moreover, exposure to higher levels of pollutants has been associated with faster cognitive decline in individuals already diagnosed with dementia (Abolhasani et al. 2023; Béjot et al. 2018; Cipriani et al. 2018). While previous reviews have focused on the epidemiological association between air pollution and dementia, few have integrated toxicological perspectives to examine the biological mechanisms underlying these associations. This review bridges these gaps by combining epidemiological evidence on exposure to air pollutants with toxicological insights into their chemical properties, transport, and biological effects. By exploring the physicochemical characteristics of air pollutants alongside their neurotoxic effects, this review aims to highlight modifiable environmental risk factors and inspire innovative prevention strategies.

This article examines major epidemiological studies on the distribution and exposure to air pollutants, particularly PM, NO₂, and O₃, while addressing toxicological findings on the biological pathways through which these pollutants exacerbate dementia progression. Given that pollutant characteristics, including their sources and distribution, significantly influence their toxicity, understanding these factors is essential for assessing their role in neurodegenerative processes. By presenting a dual perspective, this review seeks to identify actionable insights for mitigating environmental contributions to dementia.

As the global population continues to age, dementia prevention has become increasingly urgent, especially given the limited treatments available for most subtypes. Identifying and addressing modifiable risk factors such as air pollution is a critical step toward reducing the global burden of dementia.

## Criteria Pollutants and Dementia

Criteria pollutants are the most common air pollutants regulated by the Environmental Protection Agency (EPA) and serve as primary subjects of interest in this article. The EPA has established National Ambient Air Quality Standards (NAAQS) for six criteria air pollutants: PM, NO_2,_ O_3_, carbon monoxide (CO), lead, and sulfur dioxide (SO_2_). Local, state, and national agencies work collaboratively to meet and maintain these standards (Solomon 2011).

This section focuses on four of the six criteria pollutants—PM, NO_2,_ O_3,_ and SO_2_—due to their well-documented epidemiological associations with the incidence and progression of neurodegenerative disease. Although some studies have explored links between lead or CO exposure and dementia, results have been inconsistent, and further research is needed to establish stronger evidence.

For each of the pollutants discussed, we will first review their major sources and global distribution patterns, as pollutant levels vary widely across regions and seasons. Understanding these variations is crucial for developing region and source-specific mitigation strategies. We will also examine their chemical characteristics, such as composition, morphology, size, and reactivity, as these properties influence their biological effects.

To establish the relationship between these pollutants and dementia, we will summarize findings from both short- and long-term epidemiological studies. Furthermore, toxicological evidence will be reviewed to explore how specific properties of these pollutants interact with biological systems to induce neurodegeneration. By linking pollutant characteristics to their toxic effects, we aim to uncover mechanisms that explain their contribution to dementia progression and identify vulnerabilities in biological pathways.

### Particulate Matter (PM)

#### Major Sources of Atmospheric PM Pollutants

PM, including PM_10_ (≤ 10 μm) and PM_2.5_ (≤ 2.5 μm), is a mix of solid and liquid particles suspended in air, originating from both anthropogenic and natural sources. PM can be directly emitted (primary PM) or formed in the atmosphere through chemical reactions involving gaseous precursors (secondary PM) (Wu et al. 2020; Philip et al. 2014).

**Fig. 1:**
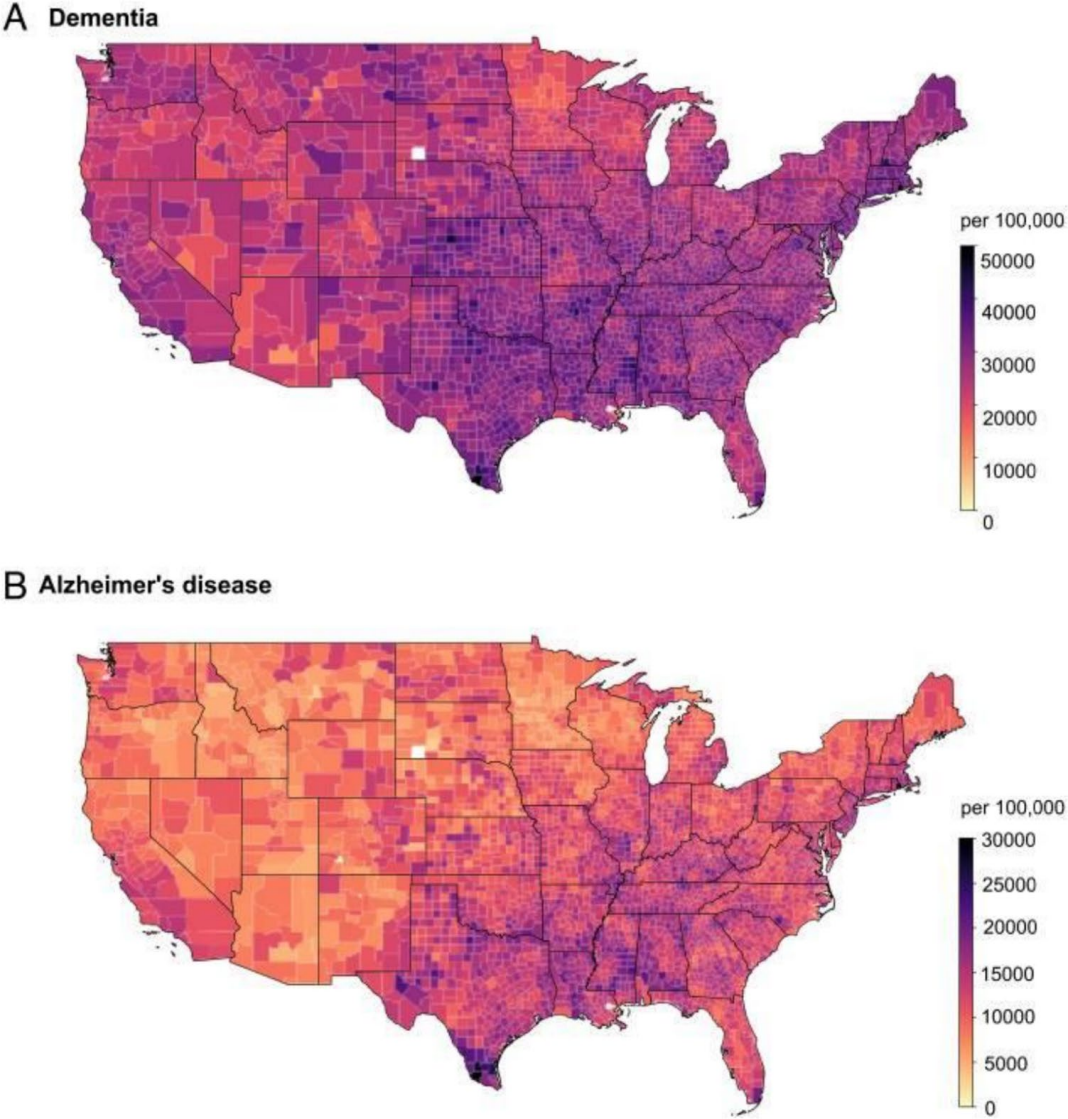
Incidence of **A** dementia and **B** AD across the contiguous United States per 100,000 Medicare beneficiaries from 2000 to 2017. From Shi et al. (2023b)

**Fig. 2:**
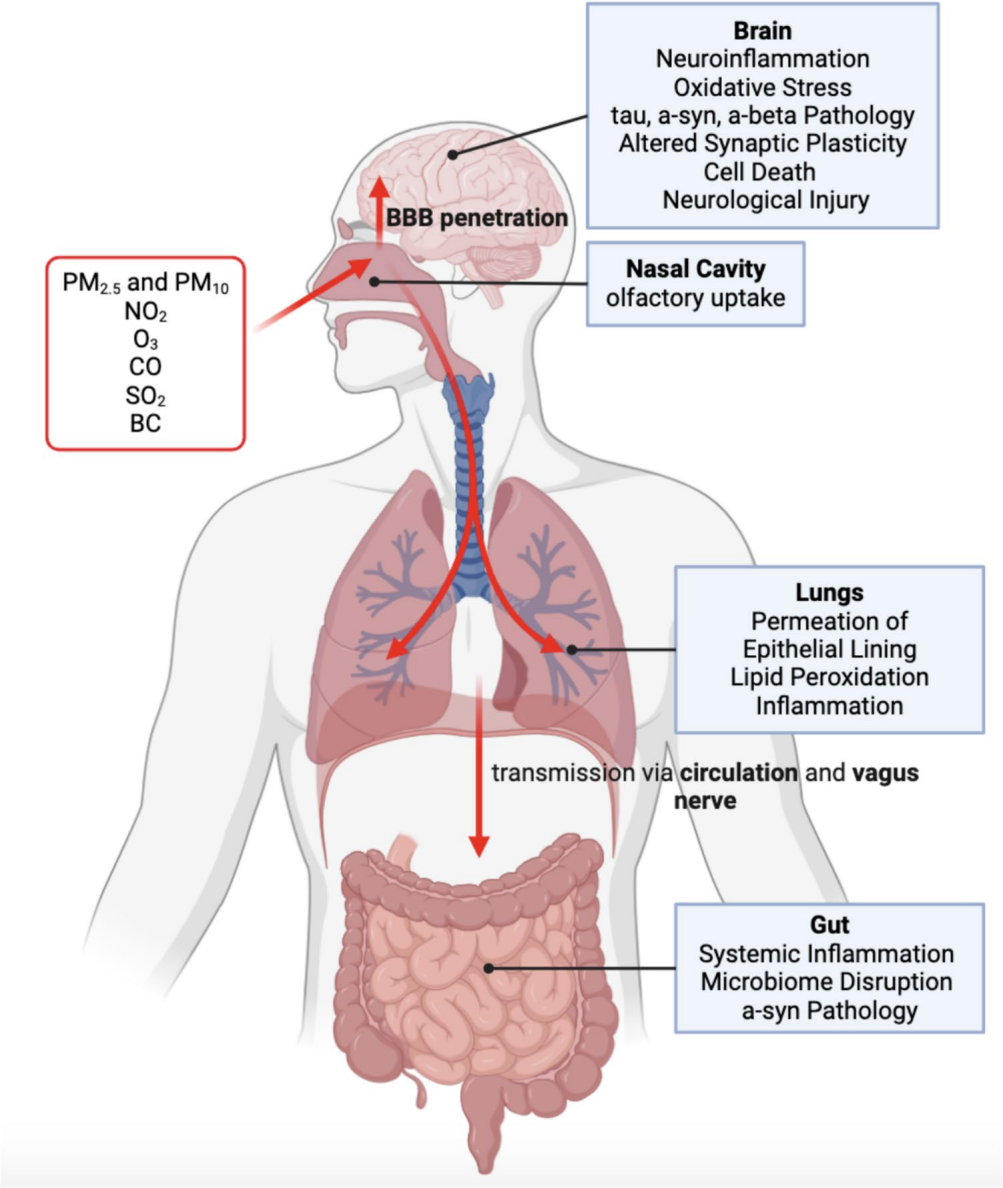
Pollutant transmission via biological mechanisms. Air pollutants enter the human body largely through the nasal cavity and are transmitted throughout various organ systems through the peripheral blood supply and penetration of organ linings. Pollutants initiate various processes in each organ system, leading to direct and indirect effects on neurodegeneration

Levels of PM and PM composition have significant geographical variability due to differences in emission sources and environmental conditions (Philip et al. 2014). Figure 1 illustrates PM_2.5_ concentrations across the USA, highlighting elevated levels in urban and industrial regions compared to rural areas (Shi et al. 2023a). Specific components of PM, such as black carbon (BC) and organic matter, are more concentrated in the southeastern USA, while nitrate, sulfate, and ammonium levels peak in the Midwest and Northeast. This regional variation underscores the importance of identifying source-specific strategies to mitigate PM pollution effectively (Shi et al. 2023a) (Fig. 2).

**Anthropogenic sources** are the most significant contributors to PM pollution globally. Combustion processes, such as vehicular emissions, coal combustion in industry and energy production, and residential burning, dominate PM_2.5_ pollution in urban areas (Philip et al. 2014). Vehicular emissions, including tailpipe and non-tailpipe emissions, dominate PM_2.5_ in urban areas. A study of PM sources in selected USA cities found that motor vehicles contributed 20–75% of ambient PM_2.5_ and 35–92% of PM_10_ (Abu-Allaban et al. 2007). Coarse PM (> 2.5 μm) in these regions primarily originated from geologic sources, such as mineral dusts, while anthropogenic fugitive, combustion, and industrial dusts can be a significant source of fine dust particles in urban locations (Philip et al. 2017). Another study across six USA cities linked PM from mobile and coal combustion sources with increased mortality, particularly when PM contained lead, sulfur, and nickel (Laden et al. 2000).

Anthropogenic sources also produce finer particles than natural sources, resulting in a higher PM_2.5_/PM_10_ ratio (Spindler et al. 2013). Combustion processes typically generate smaller particles (PM_2.5_), while mechanical processes contribute to coarser particles (PM_10_), making the PM_2.5_/PM_10_ ratio a direct indicator of emission sources and associated health risks (Mukherjee and Agrawal 2017). Diesel exhaust particles were identified as the most toxic component of PM_2.5_, followed by gasoline exhaust particles and road dust, emphasizing the critical role of on-road transportation in PM toxicity (Park et al. 2018). In addition to traffic emissions, fossil fuel combustion from residential and industrial activities significantly contributes to PM_2.5_ levels. A review of 239 source apportionment studies in China revealed that emissions from these activities peak during winter months, driven by increased heating demand (Zhu et al. 2018). Similarly, industrial emissions in Europe contribute high levels of vanadium, nickel, and sulfur oxides, as reflected in PM composition (Viana et al. 2008).

**Natural sources**, such as mineral dust emissions and sea spray, also contribute to PM, particularly in specific regions and seasons (Mukherjee and Agrawal 2017). Larger particles (> 2.5 μm) from natural sources, including soils, desert dust, and crustal materials, are often resuspended into the air by winds and other environmental triggers. For example, a global analysis identified dust as the dominant PM source in North Africa, with Saharan dust outbreaks linked to increased health risks in Europe, such as those observed in Rome, Italy (Hopke et al. 2020; Mallone et al. 2011).

In the USA, natural PM sources are predominantly associated with rural areas. In addition to natural dust emissions, anthropogenic agricultural practices and unmaintained roads significantly contribute to crustal material levels (Kundu and Stone 2014). A source apportionment study of PM_10_ in 2020–2021 identified mining, combustion sources, and windblown dust as the top three contributors (2020, 2021 Data within Australia 2020). However, urban areas within the same regions exhibited higher PM concentrations due to vehicle emissions and industrial activities.

**Open biomass burning**, including wildfires, prescribed fires, and anthropogenic agriculture burning, can emit large amount of PM and gaseous compounds, which influence regional air quality and human health (Jaffe et al. 2020). For example, wildfire emissions significantly elevate PM levels in the western USA during summer and fall (Viana et al. 2008; Hopke et al. 2020). Despite efforts to control other anthropogenic emissions, wildfire-prone regions continue to experience elevated PM_2.5_ levels. Areas affected by biomass burning demonstrate seasonally dependent variations in PM levels, exacerbated by rising temperatures, drought, and changes in wind patterns associated with climate change (McClure and Jaffe 2018; Xie et al. 2022).

#### Toxicologically Relevant Characteristics of PM

The toxicological properties of PM are influenced by its size, composition, and morphology, which vary depending on emission sources and environmental conditions. PM_2.5_ is particularly hazardous due to its ability to penetrate the respiratory tract and reach the bloodstream, delivering harmful components such as heavy metals, organic carbon, and sulfates to distant organs, including the brain (Jean et al. 2010).

#### Composition, Morphology, and Toxicity

PM_2.5_ is enriched with toxic metals like lead, nickel, cadmium, and chromium, primarily originating from anthropogenic sources such as vehicle emissions, coal combustion, and industrial processes (Tawabini et al. 2017; Srimuruganandam and Nagendra 2011). A study in Ulsan, South Korea, identified high concentrations of light metals (K, Na, Mg) and heavy metals (Fe, Zn) in coarse PM, while carcinogenic metals such as Cr, Ni, and Cd were more prevalent in PM_2.5_, contributing to higher cancer risks (Hieu and Lee 2010; Aryal et al. 2015). Similar findings were reported in Saudi Arabia, where Ba and Zn were dominant in PM_10_, while Cu and Pb were primarily detected in PM_2.5_ (Tawabini et al. 2017).

Studies in urban China and India consistently found that PM_2.5_ contained organic matter, sulfates, nitrates, and ammonium as major components (Liu et al. 2018; Srimuruganandam and Nagendra 2011). Secondary organic aerosols (SOA), formed from atmospheric reactions, further increase PM’s oxidative potential (Zhang et al. 2007). Specific components, such as vanadium, organic carbon (OC), and nitrates, were identified as the most detrimental to cell viability (Park et al. 2018).

The morphology of PM, including size and aggregation state, plays a critical role in its toxicity. Flocculent soot aggregates increase particle reactivity by adsorbing heavy metals and organic pollutants, amplifying their combined effects (Taiwo 2017; Weuve et al. 2021). Liquid-phase PM particles adhere easily to other particles, increasing their ability to penetrate biological barriers (Zhang et al. 2018; Weuve et al. 2021). Seasonal differences in PM morphology, such as higher soot content during winter, have been linked to enhanced oxidative potential and biological harm compared to summer PM (Liu et al. 2018; Zhang et al. 2018).

#### Bioaerosols and Associated Risks

Bioaerosols attached to PM include bacteria such as *Burkholderia*, *Delftia*, and *Bradyrhizobium*, which vary regionally and seasonally. These bioaerosols contribute to PM’s oxidative potential, increasing ROS production and exacerbating inflammation (Xu et al. 2019; Samake et al. 2017). A study in China reported fungal bioaerosols like *Ascomycota* as additional contributors to PM toxicity (Aziz et al. 2018).

#### Seasonal and Regional Variability

PM toxicity varies by season and region due to changes in composition. Winter PM samples often contain higher levels of sulfates, nitrates, and heavy metals like Mn, Pb, and Cu, correlating with reduced cell viability and heightened inflammatory responses (Liu et al. 2018; Zhang et al. 2018). In Hangzhou, China, residential coal combustion during winter contributed to elevated chlorine and potassium ions in PM_2.5_ (Zhang et al. 2018). These seasonal shifts have significant biological implications, with winter PM inducing earlier dose–response effects compared to summer PM.

#### Links Between PM Pollution and Dementia: Epidemiological Evidence

PM is one of the most prominent and harmful air pollutants globally, increasingly linked to the incidence of noncommunicable diseases (NCDs), including dementia (Weuve et al. 2021). While the association of PM with dementia is less studied compared to other NCDs, multiple studies have demonstrated significant correlations of AD and VaD with exposure to PM_2.5_ and PM_10_.

#### Longitudinal Studies

Recent longitudinal studies in the USA have shown that long-term exposure to PM_2.5_ is significantly associated with an increased risk of dementia and related hospitalizations. For instance, a Medicare cohort study (age ≥ 65 years) identified specific PM_2.5_ components (black carbon, organic matter, sulfate, ammonium) as strongly linked to dementia incidence (Shi et al. 2023a, 2020a, 2021a). In Seattle, a population-based study of the Adult Changes in Thought (ACT) cohort revealed that each 1 µg/m^3^ increase in the 10-year moving average of PM_2.5_ exposure was associated with a 16% higher hazard of all-cause dementia (Shaffer et al. 2021).

Additional USA studies further connect PM exposure to cognitive decline. In southwestern Pennsylvania, the MYHAT cohort study found that each 1 µg/m^3^ increase in 5-year PM_2.5_ exposure doubled the risk of mild cognitive impairment (MCI) and dementia (Sullivan et al. 2021). Similarly, in Stockholm, Sweden, long-term PM exposure over 12 + years was associated with a higher incidence of Cognitive Impairment No Dementia (CIND) and progression to dementia (Wu et al. 2022).

Studies outside the USA and Europe corroborate these findings, demonstrating a global association between PM exposure and dementia despite regional differences in PM composition. For example, in China, a 3-year study across 18 cities identified long-term exposure to PM_2.5_ and PM_10_ as risk factors for dementia and MCI, particularly among individuals with lower education levels (Tan et al. 2021). In Hong Kong, a 13-year cohort study reported a positive correlation between PM_2.5_ exposure and all-cause dementia incidence, with VaD showing a near-significant association (Ran et al. 2021). Complementing this, Li et al. specifically linked PM_10_ exposure to elevated VaD risk in a Chinese cohort. Their study found a significantly increased odds ratio for VaD with higher PM_10_ exposure 5 years prior to diagnosis. This analysis uniquely demonstrated that PM_10_ exposure contributes directly to vascular dementia risk while ruling out the contributions of other pollutants, including CO, SO₂, and O₃ (Li et al. 2019). Further evidence from Canada showed that living within 50 m of a major road—a source of traffic-related PM—was associated with an increased hazard of dementia, particularly VaD (Chen et al. 2017a). A similar study in Germany’s Ruhr district linked long-term traffic-related PM exposure to higher MCI incidence across both urban and rural settings, emphasizing the ubiquity of this risk factor (Ranft et al. 2009).

Specific to AD, multiple studies highlight PM_2.5_ exposure as a significant risk factor. A Medicare-based study demonstrated a robust association between long-term PM_2.5_ exposure and increased AD hospitalizations over 16–18 years (Shi et al. 2020b, 2021b, 2023b). The WHIMS cohort of older women across the USA found that residing in areas exceeding EPA PM_2.5_ standards increased all-cause dementia risk by 92%, with carriers of the APOE ε4 gene showing enhanced susceptibility (Cacciottolo et al. 2017). Similar findings from Taiwan identified a two to fourfold increase in AD risk among individuals in the highest tertile of PM_10_ exposure (Wu et al. 2015). The consistent association of PM exposure with dementia across diverse regions underscores the broad relevance of this environmental risk factor. In Canada, Chen et al. estimated that 6.1% of dementia cases—equivalent to 15,813 individuals—could have been prevented by reducing exposure to NO₂ and PM_2.5_ (Chen et al. 2017b). A systematic review and meta-analysis by Tsai et al. concluded that a 10 µg/m^3^ increase in PM_2.5_ exposure was positively associated with Alzheimer’s disease across multiple countries, including Canada, Taiwan, the UK, and the US (Tsai et al. 2019).

In North Carolina, Rhew et al. demonstrated that PM_2.5_ levels exceeding World Health Organization (WHO) air quality standards were associated with increased hospitalization and mortality from Alzheimer’s disease. However, the effects on PD and non-AD dementia were less pronounced (Rhew et al. 2021). Supporting this, Kirrane et al. identified a positive association between PM_2.5_ exposure and Parkinson’s disease incidence, highlighting the broader neurodegenerative impact of particulate matter (Kirrane et al. 2015).

#### Short-Term Studies

While most studies focus on long-term PM exposure, several investigations demonstrate that short-term exposure can also exacerbate dementia outcomes. In Jiangsu, China, a study found that short-term PM_2.5_ and PM_10_ exposure was associated with a 1.43% and 1.06% increase in dementia mortality hazard, respectively (Liu et al. 2022a). This underscores the immediate health risks of acute air pollution episodes. Another US-based study linked same-day PM_2.5_ exposure to increased emergency department visits for Alzheimer’s disease and related dementias (ADRDs) across five states. New York exhibited the strongest associations, emphasizing urban areas’ vulnerability (Zhang et al. 2023a).

**Policy Implications**: The evidence underscores the need for globally harmonized and stringent air quality standards to mitigate PM exposure. Targeted measures, such as reducing traffic-related emissions and controlling industrial pollutants, could significantly lower the burden of dementia and other neurodegenerative diseases (Expert Consensus Task Force et al. 2021; Sicard et al. 2021). Urban planning to minimize residential proximity to major roadways, combined with clean energy initiatives, would provide substantial public health benefits (Expert Consensus Task Force et al. 2021; Vilcassim et al. 2023).

#### PM-Associated Toxicology

PM has long been established as a respiratory irritant, causing both short- and long-term harm to respiratory health (Block and Calderón-Garcidueñas 2009; Dantzer et al. 2008, Tin-Tin-Win-Shwe et al. 2008). Increasing evidence indicates that PM also induces systemic pathological effects beyond the respiratory system, many of which are associated with dementia. These findings provide a mechanistic basis for the established epidemiological link between PM exposure and neurodegenerative diseases (Calderón-Garcidueñas et al. 2004).

#### Neuroinflammation

Exposure to PM triggers neuroinflammation through multiple pathways, which vary by particle size and composition (Block and Calderón-Garcidueñas 2009; Dantzer et al. 2008). PM can enter the brain via diffusion and active transport, stimulating immune responses and increasing the expression of pro-inflammatory cytokines. For instance, elevated levels of interleukin-1β (IL-1β), tumor necrosis factor-α (TNF-α), and chemokine ligands (CCL2 and CCL3) were observed in the olfactory bulb of mice exposed to black carbon (Tin-Tin-Win-Shwe et al. 2008). Similar upregulation of inflammatory markers, including cyclooxygenase-2 (COX-2) and CD14, has been reported in the frontal cortex and other brain regions of individuals living in highly polluted environments (Calderón-Garcidueñas et al. 2004, 2008). This inflammation was accompanied by increased Aβ accumulation, linking PM exposure to AD pathology. Importantly, PM exposure has been shown to mobilize peripheral immune cells to the brain, including bone marrow-derived neutrophils and monocytes, further amplifying neuroinflammatory responses (Mills et al. 2009). These cells migrate to the CNS, releasing cytokines and other inflammatory mediators that exacerbate local inflammation.

PM-induced neuroinflammation also involves the activation of microglia and astrocytes. Microglia transition to the pro-inflammatory M1 phenotype, releasing cytokines that activate astrocytes into a neurotoxic state, ultimately leading to neuronal death (Sama et al. 2007; Babadjouni et al. 2018; Tjalkens et al. 2017; Liu et al. 2020a). Additionally, systemic inflammation in peripheral organs, such as the liver and lungs, can amplify neuroinflammation by mobilizing cytokines like IL-6 and granulocyte–macrophage colony-stimulating factor (GM-CSF). These cytokines cross into the central nervous system (CNS), further intensifying local inflammation (Folkmann et al. 2007; Tamagawa and Eeden 2006; Peters et al. 2006; Qin et al. 2007).

#### Disruption of the Blood–Brain Barrier (BBB)

PM crosses the BBB by utilizing several mechanisms. One such mechanism is direct transport through the nasal olfactory pathway to access regions such as the olfactory bulb, brainstem, and hippocampus (Wang et al. 2008, 2007). Ultrafine PM, due to its small size, can penetrate cellular membranes and traverse the BBB (Geiser et al. 2005). PM disrupts the blood–brain barrier (BBB) through direct and indirect mechanisms, facilitating the entry of harmful substances into the CNS. Fine and ultrafine PM can penetrate the BBB, while PM exposure also increases BBB permeability by disrupting tight junction proteins, such as occludin and cadherin, via matrix metalloproteinases (MMPs) (Oppenheim et al. 2013; Calderón-Garcidueñas et al. 2014; Calderón-Garcidueñas et al. 2014; Manicone and McGuire 2008; Li et al. 2003a).

Nasal uptake of PM localizes particles in regions such as the olfactory bulb, brainstem, and frontal cortex, contributing to vascular changes and neuroinflammation (Calderón-Garcidueñas et al. 2014). PM-induced BBB breakdown has been validated in both animal models and human samples. For instance, vehicular exhaust exposure led to increased microglial activation, astrocytic reactivity, and macrophage-like cell infiltration in perivascular regions (Calderón-Garcidueñas et al. 2008; Manicone and McGuire 2008). These effects highlight the dual role of PM in damaging the BBB and initiating downstream inflammatory and degenerative processes.

#### Oxidative Stress

Under normal conditions, cells maintain a redox homeostasis, where the generation of harmful ROS by the mitochondria is balanced by antioxidant reactants. Disruption of this balance through the excess production of ROS or inhibition of antioxidant mechanisms could lead to the generation of oxidative stress (Li et al. 2003a). Increasing evidence suggests PM is capable of inducing this imbalance and triggering oxidative stress, which is highly associated with neurological disorders (You et al. 2022). Several mechanisms for oxidative stress triggered by PM have been proposed. For one, many of the components of PM, including metals and organic carbon compounds are capable of ROS generation. Thus, the composition of PM is a determinant of their ability of inducing oxidative stress. Further, intracellular localization of PM, particularly PM_2.5_, can induce oxidative stress. EM studies have shown PM is localized to damaged mitochondria, indicating their localization could have triggered mitochondrial injury and ROS production (Li et al. 2003b, 2008; Xia et al. 2004). Upon localization to the mitochondria, PM induces ROS overproduction and activation of the nuclear factor (erythroid-derived2)-like2 (Nrf2)-ARE signaling pathway. These effects cause cell death, disruptions of mitochondrial morphology, and metabolic changes (Sotty et al. 2020; Gao et al. 2022a). In vivo and e*x vivo* neurometabolic studies localize oxidative stress induced by PM to be most prevalent in the hippocampus (Park et al. 2020; Fagundes et al. 2015). Experimentally, treatment of mouse brain samples with insoluble PM resulted in much higher metabolic disturbances and ROS generation in the hippocampus in comparison to other regions such as the cerebral cortex, cerebellum, and olfactory bulb. Given the vital role of the hippocampus in AD-related dementia and memory loss, this further corroborates the role of PM in neurological disorders.

Investigations of the mechanism of action for PM-induced oxidative stress largely focus on disruption of the Nrf2 signaling pathway (Li and Nel 2006). The Nrf2 pathway is a frequently employed cellular defense pathway against oxidative stress. Guerra et al. demonstrated that chronic exposure to PM triggered this pathway (Guerra et al. 2013), whereas Chen et al. showed that the loss of Nrf2 signaling results in the inability of mice to relieve neurotoxicity induced by PM exposure (Chen et al. 2018).

### Nitrogen Dioxide (NO_2_)

#### ***Major Sources of Atmospheric NO***_***2***_

While a variety of epidemiological and toxicological studies have identified a direct linkage between PM and BC exposure and deteriorating human health, NO_2_, as a highly reactive gas, has now become a main target for many researchers’ growing interests (Atkinson et al. 2018). Atmospheric NO_2_ can be released from both natural and human activities.

**Natural sources** of NO_2_ include stratospheric intrusion of nitrogen oxides, soil emissions, bacterial and volcanic action, and lightning. However, NO_2_ of natural origin has a relatively small footprint on atmospheric concentrations as natural emissions are more globally dispersed throughout Earth’s surface (Ambient (Outdoor) Air Pollution. 2022; Fowler et al. 1998; Logan 1983). As a result, these natural sources are insignificant in impacting individuals’ health and magnifying the effects of man-made NO_2_.

52 million tons of nitrogen oxides measured in the atmosphere each year can be accredited to **anthropogenic sources (**Fu et al. 2022). Atmospheric NO_2_ is primarily resulting from the burning of fuel. During the combustion process, oxygen and nitrogen combine at very high temperature to form nitrogen oxides. In fact, only around 5–10% of emitted nitrogen oxides are in the form of NO_2_; the remainder is nitric oxide (NO). However, oxidants such as hydroxyl radicals and O_3_ can readily oxidize NO into NO_2_ in the atmosphere. Hence, NO_2_ is often considered the main proxy of nitrogen-containing pollutants (Jarvis et al. 2010). Anthropogenic NO_2_ sources can be further divided into outdoor and indoor pollutants.

According to an ensemble model of NO_2_ concentrations across the contiguous USA, NO_2_, as a combustion byproduct, is produced mainly from **outdoor sources**, specifically mobile emissions from both on-road and off-road motor vehicle exhaust. Thus, it is reasonable to find local hotspots of NO_2_ along highways and in cities (Di et al. 2020). A study of two urban areas in Romania confirms this trend in NO_2_ emissions, citing road traffic as the main source of urban NO_2_ concentrations and revealing that traffic-associated sources contribute significantly more to urban pollution than industry sources (Paraschiv and Paraschiv 2019). In addition to power plants and large fossil fuel combustors as the major sources of NO_2_ (Di et al. 2020), other minor industry-related sources include petrol and metal refineries, manufacturing facilities, and food processing sites (Li et al. 2019).

Data on indoor NO_2_ concentrations reveal that **indoor sources** of NO_2_ have an almost equal contribution to human NO_2_ exposure as outdoor sources. As of 2009, research has reported a mean NO_2_ concentration of approximately 26–112 parts per billion (ppb) in kitchens with a gas stove. In the USA, investigations of low-income households have revealed NO_2_ levels to be around 43 ppb and 36 ppb in kitchens and living rooms, respectively, and a value of 30 ppb in children’s bedrooms. For reference, the guideline for indoor NO_2_ concentration established by the WHO during study time was 21 ppb on average for 8 h (Kumie et al. 2009). Indoor NO_2_ sources can be attributed to any fuel-burning home appliances as well as tobacco smoke, further exacerbated by damaged or low quality appliances (Jarvis et al. 2010). The prevalence of NO_2_ exposure in one’s daily life calls for the pressing need to further elucidate and understand the pollutant’s impact on human health.

### ***Toxicologically Relevant Characteristics of NO***_***2***_*** Pollution***

#### Biochemical Features and Toxicity

NO_2_ is a water-soluble (Möller et al. 2019) irritant gas that is the dominating form present in air pollution among the family of nitrogen oxides (NO_X_) (Yan et al. 2016a). When inhaled, NO_2_ enters the respiratory system and irritates the respiratory tract. After reaching the lung, NO_2_ finds its way into the bloodstream through gas exchange; then it permeates through the body to different tissues and organs through blood circulation and eventually reaches the brain, where it can be neurotoxic. Memory, learning, and cognitive deficits following NO_2_ exposure reveal the detrimental effects of NO_2_ entry (Yan et al. 2016a).

NO_2_, a highly reactive oxidizer, is part of a larger category of reactive nitrogen species (RNS) (Adams et al. 2015). RNS interact with macromolecules (Adams et al. 2015) which allow them to participate in several cell signaling pathways by targeting and associating with lipids, DNA, and proteins (Martínez and Andriantsitohaina 2009). RNS’ versatility in a variety of chemical reactions, including radical recombination, addition to double bonds, hydrogen abstraction, and electron transfer (Möller et al. 2019) allows NO_2_ to play larger roles in oxidative stress and inflammatory responses (Ritz et al. 2019). Their impact on smaller-scale biological mechanisms can also be seen through their nitration of proteins and lipids, which often precedes the onset of chronic RNS-induced cellular damage (Möller et al. 2019).

#### Seasonal and Regional Variability

Among various air pollutants, NO_2_ concentrations have the highest variability temporally and spatially. Accurate characterization of NO_2_ exposure must account for local variations due to its high degree of spatial heterogeneity (Di et al. 2020). Due to its spatial heterogeneity, studies in the USA have shown that racial/ethnic minority groups are disproportionately exposed to higher levels of ambient NO_2_ pollution, indicating heterogeneity in NO_2_ exposure as an important source of environmental injustice (Wang et al. 2023; Chambliss et al. 2021; Kerr et al. 2021). The transient nature of NO_2_ concentrations can be a synergic result of many factors, some of which include chemical sources and sinks, height of the planetary boundary layer, and wind speed and direction (Di et al. 2020). NO_2_ is also subject to seasonal changes. Based on a space–time Bayesian hierarchy model monitoring hourly concentrations of air pollutants in China, NO_2_ concentrations were significantly higher during winter and autumn than spring and summer (Chen et al. 2021). Consistent with this observation, Radiello passive samplers at six sites in Cuba also showed higher levels of NO_2_ in cold seasons compared to warmer seasons (Alejo et al. 2013). This seasonal variation can be attributed to the higher hydroxyl radical levels that turn NO_2_ to nitric acid as the sink of the NO_x_ cycle during warmer months (Cichowicz et al. 2017). In addition, higher prevalence of precipitation during these months removes of NO_2_ from the atmosphere, while decreased traffic density during summer holidays also contributes to lower NO_2_ (Alejo et al. 2013). During colder seasons, however, NO_2_ levels can rise with the increased use of home furnaces and higher occurrence of smog events (Cichowicz et al. 2017).

#### ***Links Between NO***_***2***_*** and Dementia: Epidemiological Evidence***

The association between NO_2_ and dementia has been increasingly investigated throughout the years (Di et al. 2020). Analyses of different epidemiological studies around the world provide a compelling set of evidence linking NO_2_ exposure to dementia risk. Ongoing discussions surrounding NO_2_ exposure and its adverse effects on health is even more paramount given the global scale of NO_2_ exposure. However, despite a growing body of literature, such conclusions remain ambiguous due to outcome and exposure misclassifications as well as other limiting confounders, warranting future investigations and improvements.

#### Longitudinal Studies

Among earlier initiatives, a national cohort study of approximately 30,000 subjects in Taiwan was constructed to analyze the associations between NO_2_ and CO exposure and dementia risk. A longitudinal cohort study conducted by Shi et al., following the USA Medicare population from 2000 to 2018 found NO_2_ exposure is linearly associated with incidence of AD, accompanied only at a comparable level by PM (Shi et al. 2021b). Findings indicate that NO_2_ exposure is correlated with an increased risk for dementia in a dose-dependent manner, such that comparing the group with the lowest level of exposure to the highest exposure group revealed an increase of 52–56% chance for dementia occurrence. The subjects were collectively a part of the Taiwan National Health Insurance program, which provided data on their health and subsequently was able to show any onset of dementia in the randomly selected 30,000 subjects (Chang et al. 2014).

A Canadian study consisting of Quebec residents echoes these results. Using an administrative health dataset, 1.8 million people were analyzed for a period of 12 years. Exposure levels were assessed based on estimates from satellite images and ground air monitoring data which offered a geographic variation of the pollutants at a relatively small scale. The authors were able to find statistically significant associations between dementia onset and concentrations of PM_2.5_ and NO_2_. While single pollutant effects were not clearly elucidated, additional results show residential distance to major roads as another risk factor for dementia (Smargiassi et al. 2020).

Meanwhile, another Canadian study set in Ontario was able to differentiate the effects of different pollutants. Focusing on middle- to old-aged subjects who were dementia-free at the beginning of the study, Chen et al. employed 2.1 million subjects in a 12-year follow-up study. Among the three pollutants assessed, NO_2_ had the strongest influence on dementia incidence, especially on populations living near roads and highways after adjusting for diabetes, brain injury, and neighborhood income. It is also important to note that Ontario has been known to have the lowest air pollution levels in the world (Chen et al. 2017b).

However, these conclusions are in disagreement with Trevenen et al. in a similar region with relatively low concentrations of pollutants. Consisting of 111,243 men aged above 65 years, the cohort used International Classification of Diseases (ICD) diagnosis codes and study waves to diagnose incident dementia among the Australian subjects. Land-use regression models were used to estimate exposure levels at the subjects’ home address. Results reveal no association between the air pollutants—NO_2_, PM_2.5_, and BC—and incident dementia or even other subtypes of dementia including AD and VaD. This inconsistency suggests there is a potential threshold by which NO_2_ concentrations need to reach in order for an association to be observed. Additionally, a possible explanation to the discrepancy among these studies of low ambient pollutant regions is that there is not enough variability in the measured concentrations in this cohort. Taken together, it can be concluded that while NO_2_ exposure and dementia risk can be observed in relatively low average concentrations, variability may also be an important factor in detecting whether or not an association is present (Trevenen et al. 2022).

Additionally, a recent publication in Rotterdam has also cited no association between risk for dementia and determined air pollutants; neither was an association present for individual air pollutants. The initial cohort of around 8000 participants was enlarged with a second and third cohort, each containing approximately 3000 to 4000 participants. Although null findings were reported, this could be the result of assessing a relatively small study region where low variabilities in air pollutant concentrations cause significant associations to be less readily identified. Undoubtedly, a lower survival rate was observed in subjects exposed to the highest quartiles of pollutant concentrations, potentially indicating mortality reduced the onset of dementia (Crom et al. 2023).

In addition to studies of general dementia, subtypes of dementia, such as AD, PD, and VaD have also been found to have NO_2_ as a potential risk factor, although results are less consistent compared to studies grouping all types of dementia. In a Korean study of 78,830 adults from the Korean National Health Insurance Service followed for 8 years, Jo et al. concluded that the association between NO_2_ exposure and risk of incident PD was statistically significant while there were no significant correlations with the other pollutants. The authors further examined NO_2_ exposure 5 years prior to the start of the study and found aggregates of α-syn already present. This suggests the induction of a pro-inflammatory environment by NO_2_ from which α-syn formation is promoted (Jo et al. 2021).

Moreover, to assess the risk for VaD due to NO_2_ exposure, Li et al. conducted a Taiwanese study comprised 831 adults aged over 65 years with newly diagnosed VaD from Taiwan’s National Health Insurance program. After assessing the air pollution levels from air quality monitoring stations 3, 5, and 7 years before the onset of the subject’s VaD diagnosis, NO_2_ exposure was found to be significantly associated with a higher risk for VaD (Li et al. 2019).

In conflict with this study is another cohort based in London, where 130,978 middle to old age adults with no previous diagnosis of dementia were evaluated using a primary care database. While both studies were located in metropolitan regions, the latter did not produce statistically significant results for associations between NO_2_ exposure and VaD risk. Nonetheless, the London study confirmed a positive association between NO_2_ exposure and incident dementia (Carey et al. 2018).

This observation remains consistent with two cohorts conducted in the USA for over 12 million people, in which 2 million cases developed dementia while 0.8 million developed AD over an extensive follow-up period of 7 years. Administrative records from Medicare were used for outcome classifications and spatiotemporal ensemble models were used to assess NO_2_ exposures annually based on residential addresses. In both cohorts, NO_2_ was positively correlated with dementia and AD, with the latter being more strongly associated (Shi et al. 2021b).

In contrast to these outcomes, however, a French cohort study of 7,066 older adults was employed to confirm the relationship between air pollutants exposure and dementia risk. The authors found the associations between long-term exposure of PM_2.5_ and risk of all-cause dementia, AD, and incident VaD, but suggested no association with NO_2_. It is worth noting that, in contrast to other studies, this study’s protocol involved extensive follow-ups where participants with standardized questionnaires, clinical examinations, and detailed cognitive evaluation, which prevented outcome bias. However, the authors did report a possible underestimation of the association between pollutants and dementia risk, as those with higher risks of dementia as well as lower education were excluded from the study (Mortamais et al. 2021).

#### Short-Term Studies

Short-term studies corroborate this association between NO_2_ and dementia. One notable cohort based in Seoul examined the association between short-term exposure to air pollutants and PD aggravation, represented by emergency hospital admissions for primarily diagnosed PD. Similar to most studies, health statuses of participants were provided by the National Health Service while pollutant concentrations were measured hourly at monitoring sites. Findings indicate that short-term pollutant exposure likewise increases the risk for cognitive diseases, and an association with PD aggravation was found for PM_2.5_, NO_2_, SO_2_, and CO collectively (Lee et al. 2017).

Consistent with these results, Liu et al. in an investigation of 47,108 dementia-related deaths in a Chinese cohort reported an association of short-term air pollution exposure to PM_2.5_, PM_10_, and NO_2_ with dementia mortality. Exposure assessment for each individual was provided by a near-surface air pollutants dataset from CHAP (China High Air Pollutants). Results show almost 6.43% of all dementia deaths were correspondent to short-term air pollution exposure (Liu et al. 2022a).

While the single pollutant effect from these studies remains unclear, a more recent study of five USA states over a span of 10 years suggests that AD and related dementia patients may be most susceptible to the impact of NO_2_ compared to PM_2.5_ and warm season ozone. They found that increased NO_2_ levels were a significant contributor to increased emergency department visits for AD and related dementias. Specifically, this association was strongest for same-day NO_2_ exposure (Zhang et al. 2023a). Further studies are needed to examine the effects of short-term ambient air pollution exposure to a specific air pollutant to understand dementia pathogenesis and its associated risk factors.

#### ***NO***_***2***_***-Associated Toxicology***

The mechanisms by which NO_2_ exposure adversely impacts human health, as evidenced by several epidemiological studies above, have been actively studied. Promising results have shown neuronal apoptosis as one of the major cellular pathways ultimately leads to neurological diseases. This pathway, induced by NO_2_, is often considered resulting from excessive ROS production and impaired cell defense mechanisms exacerbated by a deficiency of antioxidants (Martínez and Andriantsitohaina 2009). Other investigations have specifically cited excitotoxicity and weakened synaptic plasticity as consequences of NO_2_ exposure (Li and Xin 2013; Zhu et al. 2012).

#### Mitochondrial Dysfunction and Oxidative Stress

The mitochondria and the production of ROS are often closely linked, as a prime source of ROS is the mitochondria, specifically following mitochondrial damage (Murphy 2009). Recent literature has revealed evidence linking NO_2_ exposure to mitochondrial dysfunction (Yan et al. 2015), while several previous studies have demonstrated the mitochondria’s involvement in neurodegenerative diseases and found its loss of function as a core pathological process (Wu et al. 2019). Yan et al. analyzed energy metabolism and biogenesis in rat cortices exposed to 5, 10, and 20 mg/m^3^ of NO_2_ for 7 days to deduce possible mechanisms for NO_2_-related dementia pathogenesis. Results indicate decreased expression of PGC-1α and NRF1, suggesting impairments in energy metabolism, and lower TFAM levels, which reveal disorders in mtDNA maintenance. Together, these are indicative of disturbances in mitochondrial biogenesis pathways and a decline in inner mitochondrial membrane potential which could impede ATP production. Yan et al. has also demonstrated increased ROS production subsequent to NO_2_ exposure, creating a vicious positive feedback loop that culminates in neuronal death (Yan et al. 2015). Comparable studies on rat lung and heart found NO_2_ inhalation to instigate the release of pro-inflammatory elements, such as TNF-α, IL-1β levels, and intercellular adhesion molecule 1 (ICAM-1). These activities also precede an increase in ROS, further demonstrating NO_2_-induced neurotoxicity (Yan et al. 2016a).

In AD pathogenesis, an in vivo study using C57BL/6 J and amyloid precursor protein/presenilin-1 APP/PS1 mice exposed to NO_2_ discovered the COX-2-mediated arachidonic acid (AA) metabolism of prostaglandin E2 (PGE2) as a mediator of Aβ aggravation in these mice, which is in accordance with the observed increased PGE2 levels in early stage AD patients (Combrinck et al. 2006). ROS again plays a role in this mechanism as metabolism of PGE2 is consequential to ROS attack. The study further revealed a decline in spatial learning and memory following NO_2_ inhalation (Yan et al. 2016a). Evidently, ROS continues to pose a major threat to dementia, specifically after NO_2_ exposure. Therefore, targeting the mitochondria and ROS production can be promising strategies to mitigate dementia pathology.

#### Cell Death

In addition to the mitochondrial dysfunction, other pathways of neuronal cell death caused by NO_2_ exposure have also been proposed. Results of a study by Li et al. show increased apoptosis after 7-day NO_2_ exposure in Wistar rats. Specifically, antioxidants activities were altered, which intensified the oxidative stress and raised the level of protein carbonyl (PCO), which is used as a biomarker for protein and lipid oxidative damage. Further, genes associated with cell cycle and execution of apoptosis were also expressed more abundantly (Li et al. 2012). Indeed, these results suggest that the malfunctioning cell defense mechanism is strongly correlated with the brain dysfunction and dementia upon NO_2_ exposure (Martínez and Andriantsitohaina 2009).

#### Synaptic Plasticity

Synaptic plasticity plays a major role in memory and brain functionality. Dementia is often characterized by cell death, and synaptic pathology has been one of the main contributors to disease progression (Li and Xin 2013). Many studies have also cited incident dementia being associated with ischemic stroke patients, suggesting that stroke survivors are more likely to develop dementia due to cerebral hypoperfusion (Desmond et al. 2002). A study by Zhu et al. suggested changed blood rheology properties similar to those of ischemic stroke patients’ can be seen following 5 mg/m^3^ NO_2_ exposure in healthy rats. Following this study, the researchers used the MCAO method to set up ischemic stroke model rats and revealed that NO_2_ exposure was associated with delayed neurological recovery and worsened functional recovery. These findings show NO_2_ as an inducer and promoter of ischemic stroke (Zhu et al. 2012). Ischemic stroke is an important factor in this discussion as it often precedes the onset of VaD. Research has shown a 25–30% chance in developing VaD after the occurrence of an ischemic stroke (Kalaria et al. 2016). Thus, comparing synaptic plasticity between healthy mice and mice with stroke following NO_2_ exposure may elucidate NO_2_-induced toxicological pathways that cause dementia.

To ensure that both preclinical stages—healthy or after stroke—of dementia are considered, Li and Xin analyzed the hippocampi of both healthy and stroke model rats after NO_2_ exposure and successfully verified NO_2_ as a potential risk factor for VaD. In doing so, changes in the expression of several structural markers, including synaptophysin (SYP), postsynaptic density protein 95 (PSD-95), and other long-term potentiation (LTP) related proteins, have been identified in both stroke and healthy rats. Albeit different changes, this demonstrates that in stroke model rats, NO_2_ inhalation can inhibit certain roles of proteins, posing a rather dangerous threat to synaptic function in the brain. Meanwhile in healthy rats, these changes suggest that NO_2_ exposure can perpetuate excitotoxicity in cells as increased activation of these proteins will trigger frequent miniature excitatory postsynaptic currents and ultimately lead to neuronal death (Li and Xin 2013).

Together, changes in these protein levels indicate the destructive nature of NO_2_ to brain synapses. While the current study provides evidence linking NO_2_ to VaD specifically, future approaches should aim to find underlying mechanisms for other specified types of dementia (Li and Xin 2013).

#### Insulin Signaling Pathway

The neuronal insulin signaling pathway is a key regulator for tau function. Numerous studies have characterized tau as a major hallmark of a broad range of neurodegenerative diseases. Its pathological features have been recognized in what is known as tauopathies. In a study investigating the effects of air pollution on tauopathy, Yan et al. observed that NO_2_ attenuates insulin signaling in skeletal muscle, the liver, and the brain. This triggers tau phosphorylation, driving the development of dementia (Yan et al. 2016b).

### Ozone (O_3_)

#### ***Major Sources of Atmospheric O***_***3***_

Long-term exposure to O_3_ has long been implicated to cause adverse health effects and increased mortality rate in humans (Singh et al. 2022). Along with other pollutants—including NO_2_ and PM—O_3_ levels continue to rise in populated areas, making it a pressing issue pertaining to public health (Liu et al. 2020b).

Within the lens of human health, O_3_ refers to tropospheric ozone pollution. The troposphere is the lowest level of the atmosphere where hazardous air pollutants reside (Rowland 2006). The ground-level ozone is distinct from the stratospheric O_3_, which refers to the protective layer in the stratosphere that shields the Earth from damaging ultraviolet (UV) radiation (Rowland 2006). While stratospheric O_3_ is formed **naturally** through the interaction of O_2_ with UV radiation, tropospheric ozone is produced largely from human-driven emissions. Tropospheric O_3_ is generated photochemically from reactions between nitrogen oxides (NO_x_) and volatile organic compounds (VOCs) in the presence of sunlight and heat (Sicard 2021). Methane (CH_4_) and CO can also contribute to the production of O_3_. Among the main precursors involved in O_3_ production, NO_x_ and CO are primarily released into the atmosphere by anthropogenic activities, while VOCs can originate from both biogenic and anthropogenic sources (Sicard 2021). Globally, biogenic emissions are the largest contributor of VOCs (Guenther et al. 1995).

**Anthropogenic sources** of these VOCs and NO_x_ emissions include motor vehicles, industrial plants and facilities, and man-made structures. NO_x_ is primarily produced as a result of energy consumption (e.g., electricity, heating, and transport) through fossil fuel combustion (Zhang et al. 2019). Anthropogenic VOCs are primarily generated from vehicle exhaust, gaseous fuel combustion, industrial solvents, and consumer products using volatile chemicals (Zhang et al. 2019). An EPA report assessing emission breakdown from mobile sources (vehicles) versus stationary sources found mobile and stationary sources are equally responsible for NO_x_ emission, while stationary sources are largely responsible for VOC emission and mobile sources are largely responsible for CO emission (Zhang et al. 2019). With the progression of urbanization and climate change, tropospheric O_3_ levels continue to rise in the vast urban areas of the world.

### ***Toxicologically Relevant Characteristics of O***_***3***_*** Pollution***

#### Biochemical Features and Toxicity

O_3_ is an inorganic and water-insoluble gas found in the Earth’s atmosphere (Zhang et al. 2019). While not directly emitted into the air, it is the photochemical product of interactions between NO_x_ and VOCs under the solar radiation. Tropospheric O_3_ is produced in the reaction between O_2_ and O; this highly reactive O is generated by the photolysis of NO_2_ (Zhang et al. 2019). O_3_ can be consumed through reactions with NO, giving rise back to NO_2_. Eventually a steady state level of O_3_ is established through this NO_x_ cycle. The atmospheric oxidation of VOCs and CO, however, produces free hydrogen oxide radicals that competitively bind to NO, allowing for an accumulation in O_3_ levels. As carbon emissions continue on their current trajectories, the Intergovernmental Panel for Climate Change (IPCC) projects a cumulative increase of 2 °C by the end of the century. O_3_ is generally classified as a “summer pollutant,” with reactivity of VOCs and NO_2_ being exacerbated at higher temperatures (Placet et al. 2000). Thus, global warming will continue to exacerbate the health impacts of O_3_ pollution.

ROS are a group of highly unstable compounds containing oxygen and can easily react with cellular compounds; these include the highly reactive hydrogen peroxide (H_2_O_2_) and hydroxyl radical (OH^−^) (). Such reactivity presents a possibility of cellular damage—especially to DNA, RNA, cellular organelles, and proteins. O_3_, upon reacting with biological compounds, is known to induce ROS to the cellular environment (Sies and Jones 2020). The buildup of O_3_ as a pollutant, therefore, poses the possibility of causing major damage upon entry to the human body. As a highly reactive species, O_3_ presents a threat to biological organisms when high rates of cumulative exposure to the pollutant occur.

#### Biological Uptake

O_3_ enters the body through the respiratory tract when an organism inhales ambient air with O_3_. Due to its insoluble nature, the pollutant persists in the inhaled air through the upper respiratory tract and progresses to the lower respiratory tract (Bocci et al. 1998). Water-soluble pollutants are more easily filtered from inhaled air in the upper respiratory tract before the air reaches this more vulnerable region of the respiratory system. From the lower respiratory tract, O_3_ then diffuses into the lungs through epithelial lining fluid, where it predominantly reacts with proteins and lipids found in the inner lung lining to trigger harmful cellular pathways. Research has indicated that this O_3_ reactivity in the lungs can result in the activation of inflammatory cells and stimulation of epithelial cells and macrophages to generate an inflammatory environment (Mudway and Kelly 2000). Inflammatory cell activation will lead to the formation of unstable ROS, resulting in damage to cell systems. These processes additionally result in increased permeability of the epithelial lining, leaving the lower respiratory tract even more vulnerable to pollutant entry (Mudway and Kelly 2000).

#### Regional Variability

The Tropospheric Ozone Assessment Report (TROA) consists of a database of global O_3_ measurements dating to the 1970 s (Sicard 2021). This report provides a cohesive view of tropospheric O_3_ levels across the globe; the distinction between rural and urban levels can be instructive in comparing different epidemiological studies on the O_3_ health impact. Analysis of TROA data indicates that since 1990, O_3_ has increased in urban areas at a rate of 0.31 ppb per year, and decreased in rural areas at a rate of 0.23 ppb per year (Sicard 2021). Thus, those in major cities may face an increasing risk of O_3_ exposure. Cumulative O_3_ exposure—a metric conveying overall exposure to indoor and outdoor O_3_—tends to be primarily driven by frequent and low-level indoor exposure to O_3_. Indoor O_3_ levels fall around 25% of the outdoor concentrations, with an average concentration of 4–6 ppb (Bhalla 1999). The World Health Organization has indicated an 8-h O_3_ exposure toxicity limit of 50 ppb under STP conditions, beyond which physiological and neurological toxicity could be induced. As of 2012, approximately 30% of the USA was living in areas with O_3_ levels deemed unsafe by the WHO (Nazaroff and Weschler 2022).

#### ***Links Between O***_***3***_*** Pollution and Dementia: Epidemiological Evidence***

Epidemiological studies have demonstrated a conflicting link between living in high-O_3_ concentration regions and a greater prevalence of dementia onset, while toxicological translational research suggests that acute exposure to ozone may facilitate neuroinflammatory conditions and the onset of dementia-related symptoms (Chen et al. 2017b; Carey et al. 2018; Jiang et al. 2019; Cleary et al. 2018; Erickson et al. 2017). This is an area of continued research as evidence grows for ties between other major pollutants and dementia onset but remains unclear for O_3_.

#### Longitudinal Studies

Studies conducted in Taiwan, the US, and China suggest a link between ozone exposure and dementia (Jiang et al. 2019; Erickson et al. 2017; Jung et al. 2015). A major study in Taiwan conducted from 2001 to 2010 indicated an association between high rates of cumulative exposure to O_3_—according to EPA guidelines—and the risk of developing AD (Erickson et al. 2017). The study consisted of longitudinal data of 95,690 patients, with hourly measurements of ambient O_3_ taken by the Taiwan EPA to assess the patient O_3_ exposure. Subsequent statistical analysis between AD diagnoses and ozone exposure reveals that, for an exposure increase of 10.91 ppb O_3_, subjects in the follow-up period were 211% more likely to be newly diagnosed with AD. Ozone was found to have a greater effect, however, in those without prior diagnosis of AD or cognitive impairment (Erickson et al. 2017).

Another study in the USA sampled individuals and respective cognitive performance derived from the National Alzheimer’s Coordinating Center database from 2004 to 2008 (Jiang et al. 2019). Ozone exposure was quantified using a metric from the EPA that allowed for a comprehensive coverage across the country over this 5-year period. Results indicated that an accelerated rate of cognitive decline was observed in those exposed to higher levels of O_3_ both in the cognitively normal cohort as well as the total population. Results indicated that the effects of O_3_ exposure were more dramatic in subjects that were initially cognitively normal and had little effect on those with prior cognitive impairment. The study also indicated that subjects with one or more APOE4 alleles exhibited a more rapid cognitive decline than those without the allele who experienced comparable O_3_ exposure (Jiang et al. 2019).

A third large-scale study, conducted in China from 1998 to 2018, comprising 9,544 subjects of 65 and older, supports the findings of the Taiwan and USA cohorts (Jung et al. 2015). Proportional hazard models were used to estimate the relationship between ozone levels and cognitive function and showed that an increase of 10 µg/m^3^ in annual O_3_ exposure is associated with a 10.4% higher risk of cognitive impairment. One additional study suggested that a 10 ppb increase in annual O_3_ exposure led to the equivalent of a 3.5- to 5.3-year decline in cognitive function for older subjects (Gao et al. 2022b) and that O_3_ exposure significantly exceeding EPA guidelines is associated with deterioration in executive function and dementia hospitalizations.

In contrast to the studies above suggesting a correlation between the O_3_ exposure and dementia onset, a small number of cohorts have shown a nonsignificant, or even an inverse, association between the two (Chen et al. 2017b; Carey et al. 2018). A 2001–2013 study conducted in Ontario, Canada found no association between dementia incidence and exposure to O_3_ (Chen et al. 2017b) after observation of over 250,0000 cases of dementia. Further, a large-scale cohort study published by Shi et al., which found significant association between AD and PM and NO_2_ exposure, did not find a similar link between O_3_ and dementia after investigating the USA Medicare population over the course of 10 years (Shi et al. 2021b). A study in London from 2005 to 2013 similarly demonstrated an inconsistency in the associations between pollutant types with dementia (Carey et al. 2018). NO_2_ and PM_2.5_ had positive associations with dementia diagnoses, while O_3_ yielded a hazard ratio below 1, indicating a negative association with dementia rates.

#### Short-Term Studies

The effect of O_3_ exposure on cognitive deterioration in older subjects without prior cognitive impairment and effects of short-term exposure to O_3_ remain inconclusive. Nevertheless, as the surface O_3_ level continues to rise as a result of rising anthropogenic emissions, this effect of O_3_ exposure on older subjects will likely grow more prevalent. Elucidation of potential mechanisms by which O_3_ triggers neurodegeneration could prove useful in fully understanding the mechanism of harm and possible preventative measures against this cognitive deterioration.

#### ***O***_***3***_***-Associated Toxicology***

Research into the mechanisms by which O_3_ contributes to neurodegeneration is ongoing. Following respiratory exposure, O_3_ diffuses through the mucosal epithelial lining of the lower respiratory tract to enter the body (Iaccarino et al. 2021). From there, the pollutant triggers a slew of cellular inflammatory and damage pathways that contribute to downstream cognitive impairment. Research conducted in translational rodent models enables modeling of the mechanism of the neurodegenerative damage caused by O_3_ to inform corresponding treatment.

#### Oxidative Stress

Research has indicated that a spike in oxidative stress—marked by rising ROS and lipid peroxidation (LPO) levels—and the subsequent changes in neurogenesis is often observed in the pathology of neurodegenerative disorders including PD and AD (Bromberg 2016). This oxidative stress compromises the antioxidant defense mechanism, making the brain vulnerable to damage pathways to drive the pathology of neurodegeneration.

LPO refers to the process by which oxidants react with lipids– specifically, those containing C–C double bonds (Rivas-Arancibia et al. 2010). O_3_ is especially conducive to generating ROS, which in turn react with the lipids and proteins composing the epithelial lining of the lower respiratory tract upon initial entry to the body. Thus, LPO functions as an indicator of oxidative stress in the cellular environment (Dorado-Martínez et al. 2001). Rivas-Arancibia et al. indicated that exposure of adult rats to O_3_ for extended periods**—**15 days, 30 days, 60 days, 90 days**—**was directly correlated with LPO of the rat hippocampus (Bromberg 2016). Research examining the effects of a range of ozone exposure**—**0.0, 785.57, 1374.74, and 2160.31 mg/m^3^**—**demonstrated that doses upwards of 785.57 mg/m^3^ result in elevated lipid peroxidation levels in the frontal cortex, hippocampus, striatum and cerebellum (Rivas-Arancibia et al. 2010). Similar to ROS, LPO levels are a hallmark of oxidative stress; examining resulting inflammatory stimulation can continue to delineate the pathology of neurodegeneration.

#### Neuroinflammation

Neuroinflammation has been implicated in virtually all neurodegenerative diseases and has been shown to contribute to neuronal death and neurological damage (Ayala et al. 2014). Changes in cytokine levels, increased microglial activity, and oxidative stress are factors that contribute to this inflammatory environment and have been shown to be associated with exposure to O₃, demonstrating a link between the pollutant and cognitive disease onset (Cleary et al. 2018; Rivas-Arancibia et al. 2010).

Cytokines are a class of small secreted proteins that facilitate interactions between cells, especially with regard to inflammatory pathways (Zhang et al. 2023b). Microglia are immune cells that facilitate brain development and neuronal defense in the CNS. In an analysis of cytokine levels in the cerebral cortex of mice, IL-1α and IL-13 were shown to decrease in response to O_3_ exposure, while only/keratinocyte-derived chemokine/CXC motif chemokine ligand 1 (KC/CXCL1) were shown to increase (Cleary et al. 2018). Evidently, the model did not generate a traditional neuroinflammatory mechanism with ubiquitous cytokine activation. Another study indicated that exposure to O_3_ over a 90-day period leads to increased microglial activity and phenotypic microglial changes; this is likely mediated by oxidative stress induced by O_3._ Similarly, morphological alterations of astrocytes are suggested to occur in response to oxidative stress (Zhang and An 2007). The same oxidative stress was implicated in the activation of kinases, which accelerates cytokine transcription (Zhang and An 2007).

Work by Wiegman et. al. suggests that O_3_ may induce activation of the receptor protein toll-like receptor 4 (TLR4), which in turn signals the pro-cytokine transduction pathways that activate mitogen-activated protein kinase (MAPK), NF-κB, and activator protein 1 (AP-1) (Bello-Medina et al. 2019). The NLRP3 inflammasome complex—another factor in the generation of a pro-inflammatory environment—is also shown to induce inflammation in response to mouse exposure to O_3_ (Bello-Medina et al. 2019). It is likely, then, that changes in microglia and production of cytokines contributes to the mechanism of neurodegeneration. The binding of O_3_ to receptor-like lipids and proteins in the mucosal lining of the lungs triggers an inflammatory state which ultimately results in the transmission of pro-inflammatory factors across the BBB and fosters neuronal degeneration (Cleary et al. 2018).

#### Serum amyloid A Generation

Serum amyloid A (A-SAA) is a pro-inflammatory factor derived from the liver, comprising two isoforms—SAA1 and SAA2 (Cleary et al. 2018). A-SAA has been shown to activate the generation of pro-inflammatory cytokines in microglia and astrocytes, thus creating an inflammatory environment. SAA undergoes dramatic elevation in response to infection and the subsequent host response mechanism. In AD patients, deposits of SAA were found in myelin sheaths, concentrated in the parenchyma and general neurovasculature of the brain. SAA has been found to be elevated in mouse models after O_3_ exposure.

SAA is associated with neutrophil and macrophage recruitment in the lungs, with the capability of crossing the BBB, making it a versatile target in the lung-brain mechanism of AD and PD. An immunoblot of the liver targeting the mRNA of SAA1.1 indicated greater SAA expression in mice exposed to O_3_; therefore, A-SAA is impacted by O_3_ entry into the body and subsequent creation of an inflammatory environment. Western blotting was subsequently used to confirm the same elevation in the cortex of the brain; elevated A-SAA levels in the O_3_-exposed cohort indicated the overexpression of SAA in the brain (Cleary et al. 2018).

The BBB is central in mediating the CNS environment. Capillary depletion studies of I-SAA1.1 and I-SAA2.1 established that both SAA isoforms are capable of crossing the BBB into the parenchyma. Measurement of serum SAA in brain tissue indicated that O_3_ exposure promotes the entry of A-SAA to the brain. While the direct mechanism by which O_3_ activates production of SAA is unknown, it is evident that the elevated SAA resulting from O_3_ exposure contributes to neurological inflammation and damage in the brain. SAA activation of cytokines is strongly associated with neurodegeneration as well (Cleary et al. 2018).

### Sulfur Dioxide (SO_2_)

#### ***Major Sources of Atmospheric SO***_***2***_

Like many other pollutants, SO_2_ is generated from both natural and anthropogenic sources, but anthropogenic emissions account for the major portion of SO_2_ emissions.

**Natural sources** of SO_2_ include the emission of sulfur compounds from microbes in oceans as well break down of sulfur containing organic material in forests. Both these processes release various sulfur compounds that are oxidized to SO_2_ in the atmosphere. Further, in some areas, volcanic emissions make a significant contribution to SO_2_ levels.

The main source of **anthropogenic emissions** is combustion of gasoline and diesel fuels, i.e., vehicle emissions. Based on the method of processing of specific fuels, SO_2_ may be released directly from processing plants, during fuel generation (Wiegman et al. 1957).

#### ***Toxicologically Relevant Characteristics of*** SO_2_***Pollution***

SO_2_ is colorless, reactive, and has a strong odor (Humans 2016). It is commonly produced by the metallurgic industry (Sulfur Dioxide Effects on Health 2023). Sources have also cited the use of SO_2_ in tracing levels of power plant emissions (Lee et al. 2017). Aside from the neurological impact of SO_2_, there are several other health effects resulting from SO_2_ exposure, including damages to the respiratory system causing inflammation in the lungs (Fu and Yung 2020). In addition, SO_2_ can be oxidized in the atmosphere to produce sulfate as a major component of fine PM. Here we limit our discussion to SO_2_ and the effect of PM has been discussed in Sect. “Particulate Matter (PM)”, Particulate Matter.

#### ***Links Between*** SO_2_***Pollution and Dementia: Epidemiological Evidence***

The discussions herein are only limited to the direct impacts of gaseous SO2, while SO2 can be readily oxidized in the atmosphere to produce sulfate, which is a major component of PM pollution and contribute significantly to its associations with dementia (Shi et al. 2023b). Few epidemiological studies have examined the impacts of SO_2_ and dementia with nearly all being longitudinal; with the limited results, findings have been varied and inconclusive. Within the different types of dementia, PD, VaD, and AD have been the most prominently investigated. In a cohort study in a city of South Korea assessing the association between exposure to multiple pollutants, including SO_2_, and risk of incident PD among 78,830 adult participants without PD through an 8-year follow-up, results indicated that an association between NO_2_ and dementia but insignificant association between SO_2_ and dementia (Jo et al. 2021). In contrast, a similar study conducted in Korea that investigated the impact of short-term exposure to air pollutants on the risk of PD found significant associations for all pollutants, including SO_2_. The study, conducted by Lee and her team, measured the risk for PD by analyzing emergency hospital admissions through the National Health Insurance Service-National Sample Cohort (Lee et al. 2017). Perhaps such inconsistencies in the findings are results of different study designs and methods of measuring exposure to air pollutants.

Further studies analyzing the risk of dementia and SO_2_ have continued to echo mixed results. A study conducted in Taiwan examined 831 newly diagnosed VaD cases and measured their past exposure levels to various pollutants, including SO_2_, from 76 fixed air quality monitoring stations. The results of the study suggest that there is no significant association between exposure to SO_2_ and an increased risk of VaD (Li et al. 2019).

In a similar study investigating the association between air pollution and cognitive deterioration of AD, 704 previously diagnosed AD patients were examined as well as 16 years of air pollution data were retrieved. AD cognitive deterioration was evaluated through a three-point scale. Interestingly, the results showed that cognitive decline in AD was most strongly associated with exposure to SO_2_ (Li et al. 2014). The contrasting results from different studies highlight the complexity of the relationship between SO_2_ exposure and the risk of developing dementia. Possible explanations for the inconsistency of the results could be differences in the study design, sample size, exposure assessment, and the dementia subtype under investigation. Moreover, confounding factors such as age, gender, education level, lifestyle factors, and genetic susceptibility may also contribute to the variations in findings.

Further research is required to determine epidemiological effects of short-term exposure to SO_2._

#### ***SO***_***2***_***-Associated Toxicology***

The underlying mechanisms by which SO_2_ impacts an individual’s health have not been completely elucidated; however, current findings suggest neurological disturbances are associated with SO_2_ exposure.

#### Synaptic Plasticity

In a study examining the effects of SO_2_ inhalation, male Wistar rats were exposed to two different concentrations of SO_2_ or filtered air for 90 days while the effects on spatial memory and neurobiological factors in the hippocampus were observed. Results showed SO_2_ exposure impaired spatial memory retention and decreased the expression of activity-regulated cytoskeletal associated gene (Arc) and glutamate receptor subunits (GluR1, GluR2, NR1, NR2A, and NR2B). Additional findings indicate that memory kinases and inflammatory cytokine release were also affected. Researchers were able to demonstrate the effects of SO_2_ on both behavioral and neurological degrees (Lin et al. 2022).

#### Mitochondrial Dysfunction and Cell Death

Further research has also demonstrated the joint effects of SO_2_, NO_2_, and PM_2.5_ on mitochondrial function, including reduced ATP levels, altered mitochondrial proteins, and changes in their shape. Exposure to these pollutants together impairs spatial learning and memory and alters the expression of genes related to cell death including p53, bax and bcl-2 (Yao et al. 2015).

To minimize discrepancies across studies and arrive at a more robust conclusion, it is crucial to conduct large-scale studies with standardized protocols for exposure assessment and diagnosis of dementia subtypes. A compilation of the epidemiological studies discussed in this review, with specifications of the study cohort and associated dementia subtypes is provided in Table S2. Additionally, future studies could explore the interaction between air pollution and other environmental or lifestyle factors that could affect the risk of dementia. Such an approach could provide more accurate estimates of the risk associated with SO_2_ exposure and inform public health policies to mitigate the adverse effects of air pollution on brain health.

## Other Pollutants

### Specific PM species: Black Carbon (BC) and Metals

While the effect of BC exposure on dementia and associated symptoms has been less well-established, present evidence suggests exposure to BC is associated with an elevated risk for dementia. Reducing BC emissions, thus, might be an effective method to lower rates of dementia incidence in highly exposed populations (Ku et al. 2016). Metals most commonly found in air pollutants, such as lead, mercury, and cadmium, pose significant health risks due to their association with industrial activities and vehicular emissions (Atkinson et al. 2015; Antoniadou et al. 2020).

#### Sources of BC and Metals

BC is a primary carbonaceous aerosol component of PM_2.5_, often referred to soot, and is named as such for its light absorbing properties (Dhapola et al. 2024; Andreae and Crutzen 1997). BC is largely formed from the incomplete combustion of fossil fuels and biomass through both natural and anthropogenic means (Black Carbon 2023). This includes anthropogenic combustion of coal and oil for energy generation, as well as the burning of biofuels such as wood and waste materials. Other biomass combustion occurs through various sources including forest wildfires, peat fires, and the burning of agricultural waste (Ramanathan and Carmichael 2008). Like several pollutants discussed previously, BC source appointment varies regionally. A compilation study conducted by the International Institute for Applied Systems Analysis in 2017 found that in most regions, the largest anthropogenic source of BC emissions was household biomass combustion with stoves and other residential combustion, including kerosene lamps (Ambade et al. 2022). In North America, Europe, Latin America, and the Caribbean, transport was an approximately equal contributor. Globally, household emissions accounted for the majority of emissions, responsible for 58% of global BC emissions, followed by transport (24%), industrial production (6%), agriculture (5%), fossil fuels (3%), large-scale combustion (2%), and waste (1%) (Ambade et al. 2022). Metals are commonly found in PM_2.5_ and are introduced into the environment through industrial waste. Thus, employees of fields producing high levels of industrial waste have a higher risk of metal-associated dementia (Antoniadou et al. 2020).

#### Toxicologically Relevant Characteristics of BC and Metals

An investigation into the adverse health effects of PM_2.5_ components revealed that EC, of which BC is a major form, resulted in the greatest increase in all-cause mortality per unit of increase in comparison to other components of PM_2.5_. While there is currently limited literature on the associations of BC with dementia, exposure to BC has been associated with a number of systemic diseases including cardiovascular and respiratory illnesses (Ku et al. 2016). Studies increasingly show that harmful outcomes induced in the periphery, such as inflammation, can lead to corresponding effects in the CNS, indicating these systemic diseases may eventually cause the development of dementia or related neurological disorders, although further research is required to establish this connection. Cadmium can enter the bloodstream through inhalation or ingestion, with limited amounts crossing the BBB in adults, primarily accumulating in the choroid plexus. Additionally, cadmium can bypass the BBB via the olfactory system, directly entering the brain and causing neurotoxic effects, including impaired neurogenesis and cognitive functions. Lead enters the bloodstream primarily through absorption in the lungs, gastrointestinal tract, and, in some cases, through the skin, with children and pregnant women absorbing it at higher rates. Once in the bloodstream, lead crosses the blood–brain barrier by mimicking calcium, accumulating in the brain and potentially causing neurotoxic effects (Klimont et al. 2017). Mercury can enter the brain primarily through the inhalation of mercury vapor, which easily crosses the blood–brain barrier due to its lipophilic nature. Once in the brain, mercury is oxidized to its inorganic form, where it accumulates and exerts toxic effects, particularly on the central nervous system (Bakulski et al. 2020).

#### Links Between BC and Dementia: Epidemiological Evidence

Currently available studies do, however, demonstrate an important role of BC in the incidence of dementia. A 2022 study by Li et al. examined the associations of various components of PM_2.5_ with dementia and found that exposure to BC and sulfates had the greatest associations with dementia incidence, with BC having a reported hazard ratio of 1.08. In fact, the authors found that dementia incidence increased linearly with increasing BC levels; unlike sulfates, no threshold was identifiable after which BC began to have an effect on dementia incidence (Foley et al. 2020). The association between BC and dementia is supported by a study conducted by Shi et al. in 2022. Shi et al. also found a greater association of PM_2.5_, including BC, exposure with AD than dementia, with BC having the largest effect on AD and dementia per ug/m^3^ increase in exposure. Utilizing two different exposure assessment methods, Shi et al. found that BC had hazard ratios of 1.123 and 1.247 with dementia and 1.227 and 1.393 with AD, both of which were significant enough to conclude an elevated risk for each following BC exposure (Shi et al. 2023b). A study of BC exposure and dementia incidence in a population from Vancouver, Canada found that BC has a hazard ratio of 1.03 in association with the incidence of PD and non-Alzheimer’s dementia (Li et al. 2022). Another direct population study of older men from the USA Department of Veterans Affairs Normative Aging Study found a nonlinear relationship between dementia and cognition, in which increase in exposure was associated with a lower score on the Mini-Mental State Examination and decreased global cognition (Yuchi et al. 2020). The Three City study conducted by Mortamais et al. with a base French population, found that BC had hazard ratios of 1.10, 1.00, and 1.47 with all-cause dementia, AD, and vascular or mixed dementia, respectively (Mortamais et al. 2021). BC has also been associated with decreased cognition outside the scope of dementia, as Suglia et al. found that exposure to BC in 10-year-old children resulted in decreased average vocabulary, composite intelligence, and scores on the Kaufman Brief Intelligence Test and Wide Range Assessment for Memory and Learning, indicating a stronger association between BC and cognition (Power et al. 2011). To this point, associations between BC and various kinds of dementia, including VaD, all-cause, AD, and PD have been identified, although it remains unclear which form presents the strongest association. Further research is required to corroborate evidence linking BC to each dementia subtype and identify the most robust correlations.

#### Links Between Metals and Dementia: Epidemiological Evidence

Epidemiological studies examining the effects of lead exposure on dementia consistently find that high lead levels in bone and blood are associated with accelerated cognitive aging, and even lead exposure at levels previously considered safe for adults can impair certain cognitive abilities (Suglia et al. 2008; Fenga et al. 2016). In PD, cumulative lead exposure, as indicated by bone lead levels, was associated with an increased risk of the disease, particularly in those with the highest quartile of tibia lead (Farooqui et al. 2017). For ALS, both occupational exposure and elevated lead levels in blood and bone were linked to an increased risk, suggesting a role for lead in its etiology (Weisskopf et al. 2010).

Additionally, although the association between blood lead levels and **AD** mortality was positive, it was not statistically significant, indicating a potential link between lead exposure and AD (Kamel et al. 2002). In a meta-analysis analyzing toxic metals and neurodegenerative diseases, levels of lead was found to be reduced in AD patients, while cadmium and mercury levels were significantly elevated (Horton et al. 2019). The discrepancies in results may be attributed to confounding factors, such as age in AD patients, which can influence the accumulation and measurement of toxic metal. Further assessment of cognitive function and cadmium in the elderly population suggests a consistent correlation between elevated blood cadmium levels and decreased cognitive performance (Xu et al. 2018; Li et al. 2018; Yang et al. 2023). Another study observed a significant association between blood cadmium levels and AD mortality in the elderly population (Sasaki and Carpenter 2022). However, one study utilizing urinary cadmium as a biomarker only suggested a potential association with AD mortality, particularly with shorter follow-up times (Min and Min 2016). Similar to the other two metals, the potential neurotoxic effects of mercury exposure on cognitive health was also examined, with one finding that older adults with higher blood ethyl-mercury levels are at a significantly increased risk of cognitive decline (Peng et al. 2017), and another showing a modestly elevated risk of AD among individuals with dental amalgam fillings consisted of mercury, particularly women (Geier et al. 2019).

#### BC-Associated Pathology

**Neuroinflammation:** While research regarding the mechanism by which BC induces CNS pathology is limited, it has been demonstrated that exposure to BC can induce an increase in circulating inflammatory factors, such as IL-6 and C-reactive protein, in mice. Systemic inflammation has been demonstrated to induce CNS neuroinflammation, an early stage of many kinds of dementia, indicating that BC may induce dementia through a systemic inflammation-initiated mechanism (Sun et al. 2015). Mouse model studies of BC exposure have shown that systemic inflammation induced by BC inhalation can cause BBB description through the disruption of signaling pathways such as the Wnt/β-catenin pathway (Niwa et al. 2008; Cheng et al. 2023). Given BC is a component of PM_2.5_, it is possible BC effects proceed through uptake and pathological mechanisms identified in relation to PM_2.5_, including induction of oxidative stress and neuroinflammation (Lawal 2017).

#### Metal-Associated Pathology

The "toxic metal hypothesis" posits that exposure to metals, such as lead, cadmium, and mercury, may contribute to the development of neurodegenerative disorders. **Oxidative stress:** These metals can induce oxidative stress, deplete antioxidants, and disrupt cellular functions, potentially leading to metabolic dysfunction, hormonal imbalances, and immune system impairment. Experimental and postmortem studies suggest that toxic metals can accumulate in brain cells, specifically astrocytes, neurons, and oligodendrocytes, contributing to the neuropathological features observed in these diseases (Shang et al. 2022).

**Iron accumulation:** Among these metals, iron has been most strongly linked to neurodegenerative disease and rapid cognitive decline. Changes in iron homeostasis and subsequent accumulation are observed in various neurodegenerative disorders. Iron homeostasis is closely tied to proteins such as tau, APP, and α-syn, which are central to ADRD and PD. Recent evidence indicates that iron accumulation can induce ferroptosis, a form of cell death mediated by lipid peroxidation (Gorini and Tonacci 2024; Gleason and Bush 2021), exacerbating neurodegeneration. Additionally, magnetic nanoparticles, particularly iron oxide nanoparticles in the form of aqueous colloids, magnetite and maghemite, have been found to aggregate in patients treated with them for biomedical applications (Masaldan et al. 2019; Yarjanli et al. 2017). This leads to iron deposition, protein aggregation, and oxidative stress (Masaldan et al. 2019; Yarjanli et al. 2017), resulting in neural tissue death similar to that observed in ADRD patients with high iron levels. Magnetite nanoparticles are also present in urban PM and can enter the brain directly through the olfactory nerve. These combustion-derived particles have been identified in a crystallized form in the brains of individuals exposed to polluted environments, further supporting the conclusion that iron and magnetite particles contribute to PM-related neurodegeneration (Gutiérrez et al. 2019).

Tissues exposed to elevated iron may be more susceptible to ferroptosis, which can be mitigated by iron-chelating agents. These agents have also been shown to improve cognitive performance (Maher et al. 2016), suggesting a potential therapeutic avenue. However, the current debate remains on whether iron accumulation is a cause or consequence of neurodegenerative disorders. The close association of iron accumulation with neurodegenerative processes indicates a complex pathology that warrants further investigation for new treatment development.

### Polycyclic Aromatic Hydrocarbons (PAHs)

#### Sources of PAHs

PAHs are aromatic polycyclic hydrocarbon structures that arise largely from **anthropogenic sources**. Specifically, PAHs result from the incomplete combustion of organic material that may be of natural or anthropogenic origin. Industrial sources of PAHs include waste incarceration, as well as production of metals, power, fungicide, insecticide, and rubber. Agriculture may also produce PAHs on a mass scale due to the burning of biomass and waste. Individual contributions air PAH highly overlap with sources of PM. These include emissions from large vehicles such as ships, aircrafts, and trains, as well as domestic emissions from burning of garbage, wood, and coal (Cerasuolo et al. 2023).

**Natural sources** of PAH include wildfires and volcanic eruptions but these are not as significant contributors to air PAH levels (Cerasuolo et al. 2023). While most PAHs arise from partial combustion, the organic source material of this combustion determines the type of PAH produced. Incomplete combustion of organic materials in urban areas leads to the formation of high molecular weight PAHs, whereas combustion of petroleum and its by-products leads to generation of low molecular weight PAHs. These are called pyrogenic and petrogenic PAHs, respectively. While microorganisms such as phytoplankton, algae, and plants are capable of generating PAHs, these are very minor contributions to atmospheric PAHs (Cerasuolo et al. 2023). There is seasonal variation in PAH levels, largely due to the variance of coal and biomass burning in colder versus warmer months. For example, during the cooler months of winter and spring, biomass burning makes up 18% and 14% of PAH production, while coal combustion makes up 33% and 24%, respectively (Patel et al. 2020). Further, the major contributors may change seasonally as well. During winter, vehicular emissions are higher, while ship emissions are higher in the spring (Patel et al. 2020). Over long periods of time, technological advancements can shift sources. For example, over time, coal combustion contributions have decreased as we shift to alternative energy sources, while vehicular emissions have increased (Patel et al. 2020).

#### Toxicologically Relevant Characteristics of PAHs

PAHs themselves are composed of at least 2 heterocyclic aromatic rings, which confer them high hydrophobicity and thus low solubility. The presence of aromatic rings makes them highly thermo stable, allowing them to be recalcitrant and highly persistent in the air. They accordingly have low vapor pressure and high melting and boiling points. PAHs have been demonstrated to be highly toxic and pathogenic. Specific PAHs have been shown to be teratogenic, mutagenic, and immune toxigenic, affecting organ systems throughout the body (Cerasuolo et al. 2023). A study conducted by Miura et al. measuring PAH concentrations over the Western Pacific Ocean from 2008 to 2015 found that PAH levels fluctuate seasonally. That is, PAH levels are highest in the winter and spring, followed by fall and then summer due to higher burning of fossil fuel, lower photodegradation, and lower diffusion in colder conditions (Patel et al. 2020).

#### Links Between PAHs and Dementia: Epidemiological Evidence

Epidemiological studies on the association between PAH exposure and neurodegenerative disease are far fewer than other airborne pollutants, specifically in elderly populations. However, a couple of studies have demonstrated a strong association between exposure to PAHs and NDD development in the elderly. A study conducted by Best et al. utilizing the National Health and Nutrition Examination Survey from 2001 to 2002 found that higher levels of urinary 1-hydroxypyrene, the “golden standard” of PAH exposure, was associated with poorer cognitive performance assessed by the digit symbol substitution test (DSST). Study subjects were individuals older than 60 in 15 different USA states, without known neurological disease, indicating PAH exposure could initiate cognitive decline (Miura et al. 2019). The Environmental Pollution-Induced Neurological Effects (EPINEF) study conducted by Cho et al. found similar results in the elderly population of the Republic of Korea. Researchers found PAH exposure was specifically correlated with cortical thinning in the frontal, temporal, parietal and insular lobes, caudate, and pallidum, and poorer verbal learning and memory function (Best et al. 2016). Studies investigating the effect of PAH exposure in other population have had mixed results. Coke oven workers are one such adult population with high exposure to PAHs, particularly 2-hydroxynapthalene (2-OH Nap) and benzo[a]pyrene (B[a]P).

Studies by Du et al. and Niu et al. investigating levels of 2-OH Nap and B[a]P in coke oven workers, respectively, found higher exposure to these PAHs measured by HPLC was inversely correlated with neurobehavioral function (Cho et al. 2020; Du et al. 2020). Specifically, 2-OH Nap exposure was associated with poorer performance on auditory memory tasks, while B[a]P exposure was associated with lower monoamine, amino acid, and choline neurotransmitter levels, both of which are associated with NDD development and neurotoxicity (Cho et al. 2020; Du et al. 2020).

Similarly, the EPINEF study from the Republic of Korea found PAH exposure was associated with cortical thinning and poor learning and memory function in cognitively healthy adults, measured by MRI. This study also found gender-specific effects, where specific brain cortices were thinner in men versus women (Best et al. 2016). Contrastingly, another study conducted by Ha et al. looking at effects of PAH exposure in participants of an oil spill clean-up found there was no significant correlation between urinary PAH levels and memory or cognitive disturbance after the spill (Niu et al. 2010). Importantly, this study was conducted in university students, indicating airborne PAH effects may be more pronounced in aged populations in whom they may exacerbate or initiate cognitive decline. Overall, studies report mixed results between no association and a significant association between PAH exposure and cognitive decline, with potential gender and age-specific effects. It is likely PAH exposure is associated with various cognitive deficits including executive function, planning, visual perception, and auditory and visual learning, although more research is required to confirm this association and identify the PAHs most closely associated with these deficits (Ha et al. 2012).

#### PAH-Associated Pathology

At the toxicological level, several mechanisms associated with NDD progression have been found to be induced by PAH exposure, including oxidative stress, peptide aggregation, inflammation, and compromising of the BBB. **Oxidative stress:** The Second Korean National Environmental Health Survey from 2012 to 2014 found a positive association between urinary PAH metabolites and serum gamma-glutamyltransferase (GGT), a marker for oxidative stress (Humphreys and Valdés Hernández 2022). Further, PAH exposure also causes hippocampal oxidative stress via a reduction in antioxidant enzymes (Ryu and Hong 2024). A mouse study of intranasal exposure to CRM28 in Beijing, China found exposure to PAH caused not only oxidative stress via ROS generation, but subsequently inflammation through microglial activation in the olfactory bulb and cerebral cortex (Khan and Jahego 2021). **Protein pathology:** An atomistic molecular dynamics study via GROMACS conducted by Kaumbekova et al. found PAHs call directly alter the structure of A $\beta$ peptides implicated in AD. That is, they can increase the $\beta$-sheet composition of these peptides by 2–10 percent and 50 percent decrease in alpha helix composition, promoting hydrophobic and H-bond interactions between peptides and consequently the formation of neurotoxic A $\beta$ oligomers (Tanaka et al. 2023). **BBB penetration:** In general, the lipophilic nature of the BBB and other membranes allows PAH to enter the brain. While our understanding of their neurotoxic effects is limited and specific to different PAHs, it is likely that PAH penetration into the brain induces pro-inflammatory and neurotoxic states in the brain that are highly associated with cognitive decline (Ryu and Hong 2024).

### Carbon Monoxide (CO)

#### Sources of CO

CO is a tasteless, colorless, and water-insoluble gaseous pollutant, produced primarily by the incomplete combustion of carbon compounds. Such compounds are generally emitted from gasoline vehicles, coal combustion, and waste incineration (Kaumbekova et al. 2022). Though the role of CO in the onset and progression of neurodegenerative disorders remains relatively unknown, epidemiological analysis and basic research indicates a potentially causal relationship.

#### Toxicologically Relevant Characteristics of CO

Similar to other pollutants, CO enters the bloodstream via diffusion through the membranes of lung alveoli or capillaries in blood vessels after inhalation (Peters et al. 2019). Once in the bloodstream, CO targets hemoglobin for binding. Due to CO’s significantly higher affinity for hemoglobin than oxygen, acute or chronic exposure to this pollutant can lead to reduced oxygen distribution to bodily tissues—also known as hypoxia (Fu et al. 2022). Reduced oxygen delivery to the brain and surrounding tissue can lead to central nervous system damage and neurologic pathology, underpinning a number of neurological disorders.

#### Links Between CO and Dementia: Epidemiological Evidence

A 2014 epidemiological study in Taiwan quantified the exposure of roughly 30,000 people aged 50 or older to pollutants NO_2_ and CO based on data from the National Health Insurance Research Database (NHIRD) of Taiwan (Chang et al. 2014). This data assigned exposure values to each subject based on the district where subjects would seek treatment for acute upper respiratory inspection. 1,720 of these subjects developed dementia during the study period. The study found that, relative to the lowest quartile of exposure, the second, third, and fourth (highest) quartiles had HRs of 1.07, 1.37, and 1.61 (Chang et al. 2014). With 95% certainty, researchers determined those with higher exposure to CO were diagnosed with dementia at a statistically higher rate.

While epidemiological studies have yielded relatively consistent results regarding the implications of excessive CO exposure on dementia, results appear mixed when considering specific neurodegenerative disorders.

A retrospective cohort study conducted in Korea found that exposure to pollutants played no statistically discernible role in the onset and progression of PD (Jo et al. 2021). This study used data from the Korea National Health Insurance Service, comprising roughly 80,000 of subjects aged 40 or older with no PD. No statistically significant increase in HR based on CO exposure was observed– only NO_2_ yielded an increase in HR.

Another study derived from the Taiwan NHIRD assessing the role of pollutants in VaD progression found no clear association or elevated HR of vascular dementia in patients exposed to greater levels of CO (Li et al. 2019). While NO_2_ was found to be strongly associated with vascular dementia onset, the other pollutants assessed—CO, SO_2_, O_3_—did not seem to play a role (Li et al. 2019).

#### CO-Associated Pathology

Though large-scale epidemiological studies assessing CO pollution are limited, there is compelling evidence to suggest that hypoxia-induced neuronal death may contribute to dementia. Further research is warranted, especially given the well-studied ties between other pollutants and neurodegeneration.

## Toxicology-Based Exposure Misclassifications

The discrepancies in results across studies can be attributed to various exposure misclassifications, leading to biased estimates of the true effects of air pollution on dementia. Various factors can contribute to these misclassifications, such as incomplete measurements, imprecise exposure estimates, the inability to account for individual mobility and time-activity patterns, and different exposure evaluation methods. For instance, outdoor measurements of ambient air pollution may not reflect indoor concentrations, which can be a significant source of exposure for individuals (Liu et al. 2022a; Jo et al. 2021). Moreover, dispersion modeling and spatially derived exposure estimates can be imprecise and may not represent the true exposure of a mobile population (Chen et al. 2017b). This problem is amplified for pollutants such as NO_2_, which have a higher degree of spatial variation, as compared to PM_2.5_ which follows a more uniform distribution (Kaumbekova et al. 2022). The limitations of regional exposure assessments, thus, can pose as a strong marker for bias, especially when patients are assumed to not have moved (Li et al. 2019). Using residential addresses to estimate exposure also does not account for changes in mobility or workplace (Mortamais et al. 2021; Pan et al. 2020). To compare these studies, it is also important to consider the differing methods by which each researcher executed their study. Some researchers used interpolation approaches from different monitoring sites as well as Land Use Regression approaches and satellite data (Chen et al. 2017b; Liu et al. 2022a; Trevenen et al. 2022; Crom et al. 2023; Carey et al. 2018; Mortamais et al. 2021; Kaumbekova et al. 2022), while others recorded the different air pollutant levels assessed by various monitoring sites (Li et al. 2019; Chang et al. 2014; Jo et al. 2021; Lee et al. 2017). Overall, it is important to consider the influence of exposure misclassifications and variance in exposure assessment on reported results when comparing studies.

## Discussion

Through the examination of epidemiological and toxicological evidence, we have shown that several air pollutants are associated with various dementia subtypes, exhibiting both shared and distinct pathological mechanisms. Among the pollutants discussed, NO_2_ and PM_2.5_ exhibit the most consistent and robust association with dementia, likely having the most contribution to its development. Conversely, metals, PAHs, and CO have fewer studies linking them to dementia incidence. This might reflect a scarcity of focused research rather than a lack of association.

Despite varying degrees of evidence, several pollutants share common molecular mechanisms. Nearly all pollutants induce oxidative stress and neuroinflammation, often via the generation of ROS and pro-inflammatory interleukins and cytokines. However, different pollutants trigger unique oxidative stress and inflammatory pathways. For instance, larger particles pollutants like PM and BC are known to compromise the BBB, consistent with their adsorption and transmission properties (Oppenheim et al. 2013; You et al. 2022; Niwa et al. 2008). Pollutants such as PM, NO_2_, and PAHs are associated with AD, PD, and/or FTD through mechanisms that include pathological protein aggregation (Mills et al. 2009; Yan et al. 2016b; Tanaka et al. 2023).

The convergence of these molecular pathways suggests several key points. Shared pathways indicate critical points of convergence where the effects of different pollutants can accumulate, leading to a significant physiological impact. Convergent pathways, such as neuroinflammation, BBB penetrance, and oxidative stress, can serve as targets for therapeutic intervention to mitigate the effects of polluted air. Even if individual pollutants might not produce significant effects, their combined impact, like the simultaneous impairment of multiple synaptic receptors or activation of multiple apoptosis-inducing factors, can lead to cell death and dementia (Sotty et al. 2020; Li and Xin 2013; Zhang and An 2007; Yao et al. 2015; Niwa et al. 2008; Ryu and Hong 2024).

Therapeutic development to combat these effects is underway. Treatments are being developed to address neuroinflammation, including antibodies to target receptors like TREM2 on microglia to modulate pro-inflammatory signals, small molecules to inhibit pro-inflammatory cytokine signaling and release or apoptotic pathways, and optimized NSAIDs to reduce systemic inflammation (Alemany et al. 2021). Antioxidant strategies are also being explored, with various molecules under investigation for targeting ROS in the CNS. These include cholinesterase inhibitors with secondary antioxidant properties, small molecules to replenish GSH, and compounds that prevent ROS generation. More recent research explores nanoparticles that mimic natural antioxidants like SOD and CAT or facilitate the delivery of other antioxidants to combat brain ROS generation (Liu et al. 2022b; Aborode et al. 2022; Cheng et al. 2024).

**Fig. 3:**
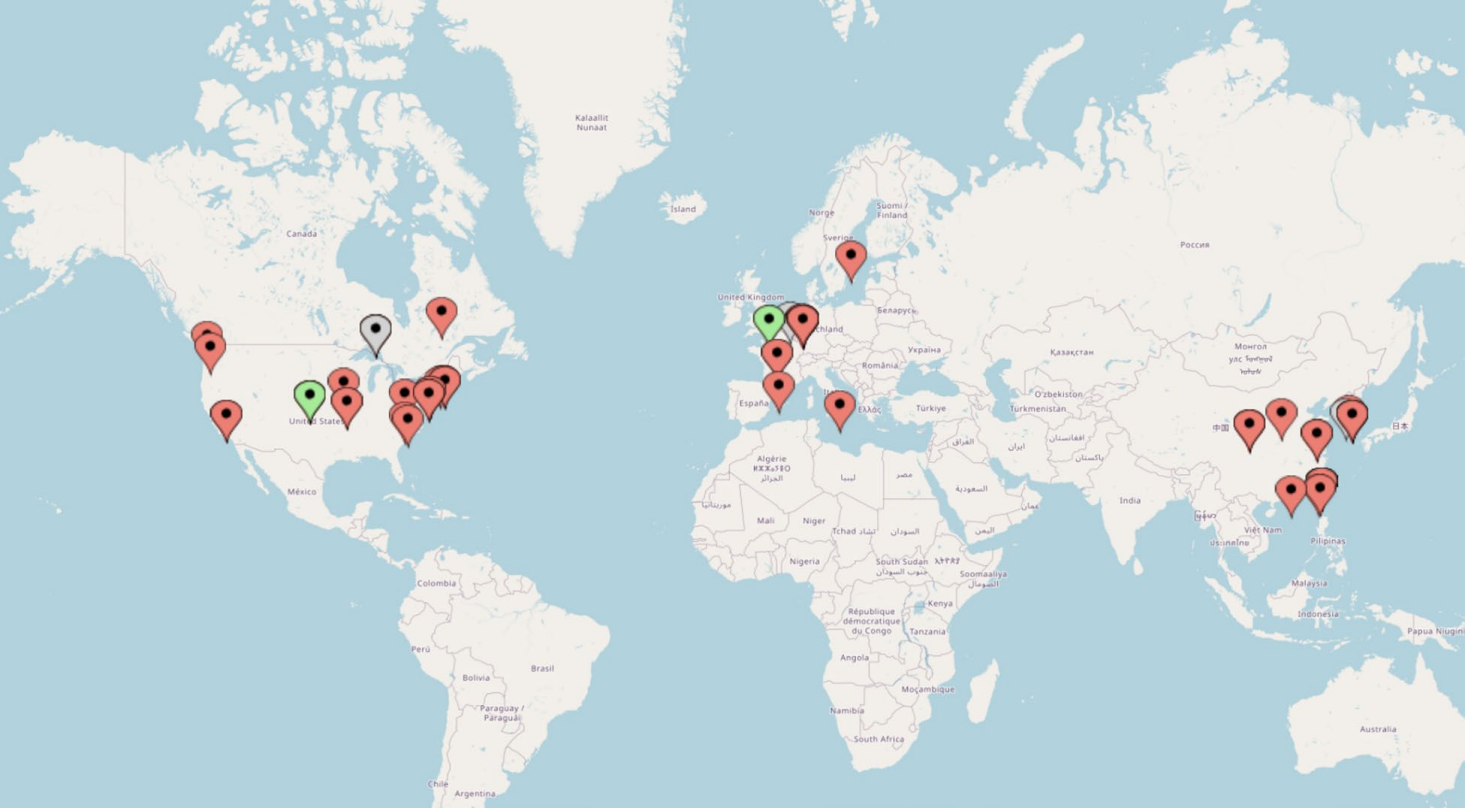
Geographical distribution of epidemiological studies included in this review. Markers on this world map represent individual epidemiological studies included in this review. Color is representative of nature of the association between air pollution exposure and dementia found in each study. Red = Significant positive association. Gray = No significant association. Green = Significant negative association. It is important to note studies vary in resolution and breadth of study population. Thus, studies whose study population included a national cohort are represented by markers in the center of the country of interest. Detailed information regarding the exposure, outcome, and study population of these epidemiological studies can be found in Table S2

The studies included in this review were conducted across a wide array of locations, and it is worth noting that environmental air pollution regulations can influence patient exposure to pollutants, potentially affecting dementia incidence. Figure 3 consolidates the results of these epidemiological studies by plotting them on a map. We observe that while studies are distributed across the US, Canada, Europe, and Eastern Asia, each with diverse air pollution regulations, exposure to pollutants was consistently associated with dementia. Exceptions where no effect was found include, the Netherlands, France, Ontario, Canada, Taiwan, and South Korea, though these were only one to two studies out of several from each location. However, it is worth noting that some of the regions where no association was found include those with the strictest ambient air quality standards (e.g., Canada with 8.8 ug/m^3^ for PM_2.5_) and that strict PM_2.5_ standards are often in areas with low population densities (Liu et al. 2021). This indicates higher regulatory standards may influence correlations between dementia and air pollution found in studies conducted in a given location by lowering overall exposure to levels that do not affect dementia incidence. Further, studies reported here are biased toward areas with low regulations due to being conducted in high density city-centered regions. Despite these exceptions, none of the countries in this review meet the WHO’s air quality standards, despite having legislative instruments to control ambient air quality, highlighting the need for more stringent regulation. A global assessment by the United Nations Environment Program revealed that 34% of countries lack legal air quality regulations, and 55% of those with regulations allow exceedances. Furthermore, 88% of countries do not have legal indoor air quality standards. This data suggests that the current level of regulation is insufficient to meet standards and control air pollution, which is likely contributing to the near-universal association with dementia. Stricter regulations could potentially mitigate this association (Nazarenko et al. 2021).

The association between dementia and air pollution might also indicate a broader connection with neurological pathology, including psychiatric disorders and mental health. Although the evidence is less robust than for dementia, there is compelling evidence linking air pollutants to these conditions like depression. A narrative review by Bhui et al., identified critical periods in children’s development during which exposure to air pollution might be particularly detrimental to their development and mental health. The review also indicate that air pollution exposure can exacerbate the deterioration of long-term conditions (First Global Assessment of Air Pollution Legislation 2021). Similarly, Zundel et al.’s systematic review found associations between exposure and depression, anxiety, and neuro- and psychopathology, including oxidative stress, neuroinflammation, and neurostructural and neurofunctional deficits (Bhui et al. 2023). Mental health research in this area is particularly focused on children and adolescents (Zundel et al. 2022), where air pollution increases the risk for suicide and psychiatric emergency department utilization (Xie et al. 2023). Many gaps in knowledge persist, including which psychiatric disorders are most associated with pollutant exposure. However, current research suggests a non-insignificant link between exposure and mental health disorders.

Despite the extensive research already conducted, several knowledge gaps remain:

- **Further Research on Understudied Pollutants**: Pollutants like CO, SO_2_, and PAHs required more research, as current studies are sparse, with many finding no association. Establishing correlation (or lack thereof) with dementia and isolating these effects from co-expressed pollutants is necessary.
- **Standardization of Outcome Measurements**: There is a need to standardize outcome measurements to confirm relationships and ensure consistency across different regions that might employ varying metrics, such as hospitalizations, dementia diagnosis, or the presence of biomarkers. Utilizing fingerprinting biomarkers for neurological disorders could aid in this standardization, thereby reducing measurement errors and enhancing the comparability of study results.
- **Air Quality Regulations and Dementia Incidence**: Additional research investigating the direct association between air quality regulation and dementia incidence is needed, following the United Nations’ comprehensive review, to validate the argument for stricter air quality regulations as a preventative measure against dementia.
- **Effective Prevention and Targeted Therapeutics**: There is a significant gap in therapeutic development concerning air pollutant-related mechanisms. While drugs targeting common pathways such as neuroinflammation or oxidative stress are under development, strategies focusing on the prevention of pollutant uptake or tailored to region/job-specific exposure profiles could be more effective, particularly in urban areas with unique pollution sources.

Articles in this study included epidemiological and experimental (biological and toxicological) studies. Epidemiological studies were published no earlier than 1995 (although a couple of these studies included data from population records older than 1995), included any geographical location across the world, had aged study populations (usually 65 years or older), and were mainly cross-sectional or cohort studies. Where these criteria were not met (i.e., when case–control studies were conducted or younger populations were studied), this was specified when the study was discussed in our review. All epidemiological studies investigated a relationship between any of the air pollutants mentioned in this article and ‘dementia,’ ‘cognitive dysfunction,’ or ‘neurodegenerative disease,’ found through a PubMed search. Keywords ‘dementia’, ‘cognitive dysfunction’, ‘neurodegenerative disease’, ‘air pollution’, in conjunction with ‘particulate matter’, ‘nitrous oxide’, ‘sulfur dioxide’, ‘black carbon’, ‘ozone’, ‘carbon monoxide’, ‘bioaerosols’, and ‘polyaromatic hydrocarbons’ were utilized in our Google Scholar and PubMed searches. Metareviews were also included where this association had already been reviewed, for some more commonly studied pollutants such as PM2.5. Additionally, basic science experimental studies were included for the toxicology sections to describe molecular routes by which each pollutant may be affected NDD development.

The strengths of this review article include the broad scope, covering criteria pollutants most strongly associated with dementia along with minor contributors. For each pollutant, we provided epidemiological evidence, physical properties, and toxicological pathogenic mechanisms, offering a comprehensive overview of pollutants’ influence on dementia. By consolidating this information, we identified research gaps for various fields, from basic scientists to epidemiologists. It is worth noting the review reported here was a narrative review with the associated limitations. The authors of this review have expertise in cellular/molecular neurodegeneration and environmental toxicology, so the organization of this review is biased toward this focus. Further, while the above criteria were used to select included studies, there was no automated or standardized process used, as seen in a systematic review. Finally, this review does not cover all relevant studies and reviews on air pollution, although the most significant in our perspective have been included. No quantitative analysis was performed, so we cannot provide numerical significance regarding which pollutants are most closely associated with dementia or specific subtypes. Additionally, while including studies from various geographic locations allowed us to identify high-level trends, region-specific influences might be overlooked, necessitating further research in specific populations.

## Conclusion

This review presents a novel, interdisciplinary, and comprehensive examination of the interactions between various types of air pollutants, dementia, and the molecular pathways potentially involved. For the first time, we have integrated studies that trace the pathway from pollutant exposure to biological outcomes. By discussing the properties of pollutants, epidemiological studies, and pathological mechanisms for each common air pollutant, we aim to provide a holistic model of the current state of research on pollutant-associated dementia. In doing so, we consolidate a wealth of studies investigating this association, highlighting novel connections between specific components and properties of air pollution, dementia subtypes, and molecular pathways.

The associations we’ve identified have far-reaching implications for a broad range of fields, including toxicology, public health, neuroscience research, as well as studies on neuroinflammation, oxidative stress, and their roles in neurological disorders. Our examination of numerous longitudinal and short-term studies has led to the conclusion that there is a clear association between air pollution exposure and the incidence, severity, and mortality of dementia. This association is most pronounced with MCI (a precursor of dementia), followed by VaD, all-cause dementia, and AD, in comparison to PD. Despite the use of varying standards for air pollutant exposure assessment and dementia diagnosis across regions and studies, several have established a robust link between the two.

Among the air pollutants examined, the association with dementia incidence appears strongest for PM_2.5_ and NO_2_. However, due to the current lack of comprehensive datasets, further research is warranted to explore potential association between dementia incidence and exposure to pollutants like black carbon, O_3_, SO_2_, PAHs, and CO, particularly to identify dementia subtypes associated with each. It is important to recognize that the impact of each pollutant on dementia incidence varies significantly by location. For instance, the effects of PM_2.5_ and PM_10_ are largely dictated by their composition and sources, which can differ greatly by region and even seasonally. Nevertheless, the established strength of association underscores air pollution as a primary target for reducing dementia incidence in any region. Supported by preclinical studies and some established mechanisms of pathological induction, the development of therapeutics and the implementation of regulations to control air pollution levels in both urban and rural areas are promising strategies to combat dementia incidence globally.

## Supplementary Information

Below is the link to the electronic supplementary material.

Supplementary file1 (DOCX 29 kb)

## Data Availability

Not applicable.
