# Supplementary material for: The Epidemiological and Toxicological Intersection of Air Pollution and Dementia
Source: Rev Environ Contam Toxicol. 2025 Oct 10;263(1):25. doi: 10.1007/s44169-025-00092-6 (PMC12513895; doi:10.1007/s44169-025-00092-6)
Supplement: Supplementary file 1 — Supplementary file1 (DOCX 29 kb) [file 44169_2025_92_MOESM1_ESM.docx]

**Supplemental Figures**

**Table 1. Pollutant-associated pathologic mechanisms, altered agents, and dementia subtypes.**

| Pollutant | Mechanism | Altered Agents | Associated Dementia Subtypes* |
| --- | --- | --- | --- |
| PM_2.5_ and PM_10_ | Neuroinflammation | IL-1β, TNF-α, CCL2, CCL3, COX-2, CD14, IL-6, GMCF  Astrocyte and microglial phenotypes (A1/M1) | AD, PD |
|  | BBB Disruption | Microvasculature proteins: occludin, cadherin, MMPs  Macrophage localization | AD, VaD |
|  | Oxidative Stress | ROS  Nrf2 Signaling |  |
|  | Pathologic Protein Deposition and Spread | A, tau, α-syn | AD, PD |
| NO_2_ | Oxidative Stress | ROS  PGC-1α, NRF1  TFAM and mitochondrial morphology/function  COX-2, AA, PGE2 metabolism | AD |
|  | Impaired Cell Defense | PCO and other markers of cell damage and apoptosis |  |
|  | Neuroinflammation | TNF-α, IL-1β, ICAM-1 |  |
|  | Neurological Damage | Ischemic stroke | VaD |
|  | Impaired Synaptic Plasticity | PSD-95, SYP, and other LTP-associated proteins  Excitotoxicity | VaD |
|  | Insulin Signaling | tau | AD, FTD |
|  | Pathologic Protein Deposition | Aβ, tau |  |
| O_3_ | Oxidative Stress & Lipid Peroxidation | ROS  LPO levels | AD, PD |
|  | Neuroinflammation | IκB phosphorylation  IL-1α, IL-13, KC/CXCL1  TLR4, MAPK, NF-κB, AP-1 pathways  Astrocyte and microglial phenotypes (A1/M1)  A-SAA | AD, PD, VaD |
| CO | Hypoxia | Blood oxygenation levels | PD, all-cause |
| SO_2_ | Inflammation | TNF-α, IL-1β and IL-6 |  |
|  | Synaptic Plasticity | Arc  GluR subunits: GluR1, GluR2, NR1, NR2A, and NR2B |  |
|  | Mitochondrial Deficits | ATP  Morphology and protein composition |  |
|  | Cell Death | p53, BAX, BCL-2 |  |
| BC | Neuroinflammation | IL-6, C-reactive protein, TNF-α, IL-1β |  |
|  | Blood-Brain Barrier Disruption | Wnt/β-catenin |  |
|  | Oxidative Stress | AhR, Nrf2 |  |
|  | Mitochondrial Dysfunction | PTEN, PINK1/Parkin  Mitophagy | PD |
| Metals | Oxidative Stress & Antioxidant Depletion | ROS | AD, PD |
|  | Metal Accumulation | Pb, Hg, Cd |  |
| PAH | Oxidative Stress | GGT  ROS |  |
|  | Pathologic Protein Deposition | Aβ | AD |
|  | Neuroinflammation | Microglial phenotypes (M1) |  |

*Empty entries under “Associated Dementia Subtypes” indicates the Mechanism mediated by the given Pollutant has not been associated with a specific subtype of dementia.

**Table 2. Epidemiological studies of air pollutant exposure and dementia.**

| Pollutant | Ref. | Modality of  Exposure Assessment | Modality of  Outcome Assessment | Study Population/Cohort | Association?* | Risk or progression of Dementia |
| --- | --- | --- | --- | --- | --- | --- |
| PM_2.5_ and PM_10_ | ^40^ | Linked spatiotemporal model-based  PM  2.5  exposures to participant addresses | Standardized, consensus-based protocols at biennial follow-ups | Adult Changes in Thought (ACT)  Seattle, Washington, USA | Positive | Risk |
|  | ^41^ | Geocoded participants’ residential address to census tract level PM2.5 from Environmental Protection Agency (EPA) air quality monitors | Clinical Dementia Rating (CDR) | Monongahela-Youghiogheny Healthy Aging Team (MYHAT) Cohory  Monongahela Valley, Pennsylvania, USA | Positive – dementia, MCI | Risk |
|  | ^42^ | Residential addressed assessed using spatiotemporal dispersion modeling based on local emission inventories and meteorological data | Swedish National Study on Aging and Care in Kungsholmen (SNAC-K) | Swedish National Study on Aging and Care in Kungsholmen (SNAC-K)  Stockholm, Sweden | Positive – dementia, CIND | Risk |
|  | ^43^ | Linked residential addresses to a machine learning method (random forest model) with ground monitoring data, satellite-retrieved aerosol optical depth, and information on other spatial and temporal predictors | Chinese Veteran Clinical Research Platform | Chinese Veteran Clinical Research Platform  China | Positive – dementia, MCI | Risk |
|  | ^44^ | Residential addressed linked to spatiotemporal PM_2.5_ concentrations using aerosol optical depth (AOD) recordings and data from general monitoring stations | Chinese Elderly Health Service (EHS) Cohort | Chinese Elderly Health Service (EHS)  Hong Kong, China | Positive | Risk |
|  | ^45^ | Residential address linked to monitoring data collected by the Taiwan Environmental Protection Administration | Taiwan’s National Health Insurance Research Database (NHIRD) | National Health Insurance Research Database (NHIRD)  Taiwan | Positive - VaD | Risk |
|  | ^46^ | Residential proximity to major roadways or highways | Ontario's Registered Persons Database | Ontario, Canada | Positive - VaD | Risk |
|  | ^47^ | Residential address linked to monitoring stations | Neuropsychological test battery CERAD-Plus | Study on the Influence of air pollution on Lung function, Inflammation, and Aging  Ruhr, Germany | Positive - MCI | Risk |
|  | ^51^ | Bayesian Maximum Entropy method based on residential address | Standardized WHIMS outcome ascertainment protocols | Women’s Health Initiative Memory Study (WHIMS)  USA | Positive - AD | Risk |
|  | ^52^ | Linked residential address to monitoring stations from the Department of Taiwan Air Quality Monitoring Network, Environmental Protection Administration | Mini-Mental State Examination | Taiwan hospitals | Positive – AD, VaD | Risk |
|  | ^53^ | Linked residential address to satellite observations in combination with outputs from a global atmospheric chemistry transport model | Cases of dementia were defined as any individual having ≥ one hospital admission with a diagnosis of dementia or three physician claims over a two-year period or a prescription relating to dementia | Ontario, Canada | Positive | Risk |
|  | ^55^ | Linked zip code to Moderate Resolution Imaging Spectroradiometer (MODIS), Multi-angle Imaging SpectroRadiometer (MISR), and Sea-viewing Wide Field-of-view Sensor (SeaWiFS) Aerosol Optical Depth (AOD) measurements | Causes of death and primary diagnoses of hospital admissions | Piedmont, North Carolina, USA | Positive – AD, non-AD dementia, PD | Risk |
|  | ^56^ | Linked residential address to hierarchical Bayesian model that combines monitoring data from the U.S. Environmental Protection Agency’s (EPA) Air Quality System (AQS) with numerical output from EPA’s Community Multiscale Air Quality Model (CMAQ) | self-reports of PD collected at enrollment and two follow-up interviews | Agricultural Health Study  North Carolina and Iowa, USA | Positive - PD | Risk |
|  | ^57^ | Extracted air pollutant concentration from China High Air Pollutants (CHAP) dataset at geocoded residential address | Based on medical records and information collected from the household | Jiangsu province, China | Positive | Progression |
|  | ^58^ | Patient zip codes linked to spatiotemporal ensemble models | Condition algorithm from the Centers for Medicare and Medicaid Services to identify AD and ADRD ED visits using both primary and secondary diagnosis codes | California, Missouri, North Carolina, New Jersey, New York, USA | Positive – AD, ADRDs | Risk |
|  | ^50^,^49^ | Linked patient zip codes to superlearning and ensemble weighted averaging models and previously validated PM2.5 composition prediction model | Centers for Medicare and Medicaid Services (CMS), including the Medicare denominator file and the Medicare Chronic Conditions Warehouse (CCW) | USA Medicare population | Positive – AD, PD, ADRDs | Risk |
| NO_2_ | ^114^ | Monitoring station present in the residential district in which the clinic where the people most frequently sought treatment for acute upper respiratory infection was located | National Health Insurance Research Database (NHIRD) | National Health Insurance Research Database (NHIRD)  Taiwan | Positive | Risk |
|  | ^115^ | Distance to major roads at their residential address according to the centroid of their postal code of residence throughout their follow up | Québec Integrated Chronic Disease Surveillance System (QICDSS) | Quebec, Canada | Positive | Risk |
|  | ^53^ | Linked residential address to satellite observations in combination with outputs from a global atmospheric chemistry transport model | Ontario Population Health and Environment Cohort (ONPHEC) | Ontario, Canada | Positive | Risk |
|  | ^117^ | participants geocoded residential address linked to land-use regression (LUR) models | Screened for dementia at Rotterdam Study | Rotterdam | Not Significant |  |
|  | ^118^ | Linked residential address to. Monitoring sites from the Seoul Research Institute of Public Health and Environment | Newly diagnosed cases of PD as individuals who were newly diagnosed with a primary or subsidiary diagnosis with an ICD-10 code for PD (G20) together with the rare intractable disease registration code for PD (V124) | Korean National Health Insurance Service  South Korea | Positive - PD | Risk |
|  | ^45^ | Residential address linked to monitoring data collected by the Taiwan Environmental Protection Administration | Taiwan’s National Health Insurance Research Database (NHIRD) | Taiwan National Health Insurance Program beneficiaries  Taiwan | Positive - VaD | Risk |
|  | ^119^ | Postcodes linked to KCLurban dispersion modeling system | Clinical Practice Research Datalink | London, U.K. | Positive - AD | Risk |
|  | ^218^ | Residential address linked to air pollution monitoring campaign of the European Study of Cohorts for Air Pollution Effects (ESCAPE | Cognitive performance falling outside the established cutoffs of the following cognitive tests: Mini-Mental State Examination, Memory Impairment Screen, Time-Oriented subset of the Barcelona Test II, semantic fluency | Alzheimer’s and Families Cohort (ALFA+)  Barcelona, Spain | Positive - AD | Risk |
|  | ^120^ | Geocoded baseline residential address of each participant using hybrid land-use regression (LUR) models | Follow-up visits performed every two years including standardized questionnaires, clinical examinations, and detailed cognitive evaluations. | Three City study cohort  France | Not Significant |  |
|  | ^121^ | Linked residential address to monitoring sites operated by the Korean National Institute of Environmental Research | Admission cases based on medical treatment of patients diagnosed with PD | Korea National Health Service beneficiaries  Seoul, South Korea | Positive - PD | Progression |
|  | ^57^ | Extracted air pollutant concentration from China High Air Pollutants (CHAP) dataset at geocoded residential address Based on medical records and information collected from the household | Death certificates with mortality from dementia | Jiangsu province, China | Positive | Progression |
|  | ^58^ | Patient zip codes linked to spatiotemporal ensemble models | Patient-level ED visits from hospital associations or state health departments | California, Missouri, North Carolina, New Jersey, New York, USA | Positive - AD | Risk |
|  | ^49^ | Linked zip codes to monitoring measurements from the Environmental Protection Agency (EPA) Air Quality Systems (AQS) | Medicare Chronic Conditions Warehouse (CCW) | USA Medicare population | Positive – AD, ADRDs | Risk |
| O_3_ | ^148^ |  |  | Taiwan | Positive - AD |  |
|  | ^146^ | 3-digit zip codes linked to US Environmental Protection Agency (EPA) data | Mini-Mental Status Examination (MMSE) and Cognitive Dementia Rating Sum of Boxes | National Alzheimer’s Coordinating Center  USA | Positive | Progression |
|  | ^149^ | Baseline county of residence linked to a machine learning model based on the eXtreme Gradient Boosting (XGBoost) algorithm | Interview administered by a well-trained interviewer and a basic health examination performed by a nurse or medical student | China | Positive | Risk |
|  | ^53^ | Linked residential address to satellite observations in combination with outputs from a global atmospheric chemistry transport model | Cases of dementia were defined as any individual having ≥ one hospital admission with a diagnosis of dementia or three physician claims over a two-year period or a prescription relating to dementia | Ontario, Canada | Not Significant |  |
|  | ^119^ | Postcodes linked to KCLurban dispersion modeling system | Clinical Practice Research Datalink | London, U.K. | Negative |  |
|  | ^49^ | Linked zip codes to monitoring measurements from the Environmental Protection Agency (EPA) Air Quality Systems (AQS) | Medicare Chronic Conditions Warehouse (CCW) | USA Medicare population | Negative |  |
| CO | ^114^ | Monitoring station present in the residential district in which the clinic where the people most frequently sought treatment for acute upper respiratory infection was located | National Health Insurance Research Database (NHIRD) | National Health Insurance Research Database (NHIRD)  Taiwan | Positive | Risk |
|  | ^118^ | Linked residential address to. Monitoring sites from the Seoul Research Institute of Public Health and Environment | Newly diagnosed cases of PD as individuals who were newly diagnosed with a primary or subsidiary diagnosis with an ICD-10 code for PD (G20) together with the rare intractable disease registration code for PD (V124) | Korean National Health Insurance Service  South Korea | Not Significant (PD) |  |
|  | ^45^ | Residential address linked to monitoring data collected by the Taiwan Environmental Protection Administration | Taiwan’s National Health Insurance Research Database (NHIRD) | National Health Insurance Research Database (NHIRD)  Taiwan | Not Significant (VaD) |  |
| SO_2_ | ^118^ | Linked residential address to. Monitoring sites from the Seoul Research Institute of Public Health and Environment | Newly diagnosed cases of PD as individuals who were newly diagnosed with a primary or subsidiary diagnosis with an ICD-10 code for PD (G20) together with the rare intractable disease registration code for PD (V124) | Korean National Health Insurance Service  South Korea | Not Significant |  |
|  | ^121^ | Linked residential address to monitoring sites operated by the Korean National Institute of Environmental Research | Admission cases based on medical treatment of patients diagnosed with PD | National Health Insurance Service-National Sample Cohort  South Korea | Positive - PD | Progression |
|  | ^45^ | Residential address linked to monitoring data collected by the Taiwan Environmental Protection Administration | Taiwan’s National Health Insurance Research Database (NHIRD) | National Health Insurance Research Database (NHIRD)  Taiwan | Not Significant (VaD) |  |
|  | ^163^ | Residential address linked to data from Taiwan Environmental Protection Administration | Annual interview with Clinical Dementia Rating | Kaohsiung and Pingtung, Taiwan | Positive - AD | Progression |
| BC | ^176^ | Patient zip codes linked to satellite retrievals of aerosol optical depth, chemical transport modeling (CTM), and ground-based observations | The Medicare denominator file and Medicare Chronic Conditions Warehouse (CCW) | Medicare Chronic Conditions Warehouse  Northeastern USA | Positive | Risk |
|  | ^50^,^176^ | Linked patient zip codes to superlearning and ensemble weighted averaging models and previously validated PM2.5 composition prediction model | Centers for Medicare and Medicaid Services (CMS), including the Medicare denominator file and the Medicare Chronic Conditions Warehouse (CCW) | USA Medicare population | Positive – AD, ADRDs | Risk |
|  | ^177^ | Residential addresses linked to land-use regression (LUR) models specific to Metro Vancouver | Diagnosis from hospital records, physician visits from MSP and prescriptions from PharmaNe | Vancouver, Canada | Positive – PD, non-AD dementia | Risk |
|  | ^178^ | Residential address linked to validated spatiotemporal land-use regression model | Mini-Mental State Examination (MMSE), the digit span backward test, a verbal fluency task, constructional praxis, immediate recall of a 10-word list, delayed recall of a 10-word list, and a pattern comparison task | USA Department of Veterans Affairs Normative Aging Study | Positive | Risk |
|  | ^120^ | Geocoded baseline residential address of each participant using hybrid land-use regression (LUR) models | Follow-up visits performed every two years including standardized questionnaires, clinical examinations, and detailed cognitive evaluations. | Three City study cohort  France | Positive – AD, VaD, mixed dementia | Risk |
| Metals | ^180^ | Environmental assessment of workplace lead levels was given by factory management and was over the threshold limit value of 0.05 mg/m3 set by the ACGIH | Branches Alternate Movements Task (BAMT), Finger Tapping (FT), Digit Span (DS, and Profile of Mood States (POMS) | University of Messina, Italy | Positive | Risk |
|  | ^181^ | Bone lead was measured using K-shell X-ray fluorescence (KXRF) spectroscopy | Clinical examination and complete health and lifestyle questionnaires including Mini-mental status exam every 3 to 5 years | VA Normative Aging Study  Massachusetts, USA | Positive | Risk |
|  | ^183^ | Collection of blood samples and measurement of bone lead | Diagnosis of ALS was based on criteria published by the World Federation of Neurology | New England  Boston, MA | Positive – ALS | Risk |
|  | ^185^ | N/A | N/A | USA National Health and Nutrition Examination Survey | Positive - AD | Progression |
|  | ^186^ | Blood samples were collected from participants by venipuncture in prescreened phials or vacuum tubes | Consortium to Establish a Registry for Alzheimer’s Disease (CERAD) Word List Learning Test, the CERAD Word List Recall Test, the Animal Fluency test and the Digit Symbol Substitution Test (DSST) | USA National Health and Nutrition Examination Survey | Positive | N/A** |
|  | ^189^ | Available blood cadmium data from Third National Health and Nutrition Examination Survey (NHANES) and the Linked Mortality File | Third National Health and Nutrition Examination Survey (NHANES) and the Linked Mortality File | Third National Health and Nutrition Examination Survey | Positive - AD | Progression |
|  | ^182^ | Bone lead measurements were taken at two anatomical sites with a KXRF instrument | Movement disorder specialists using the U.K. brain bank criteria | Boston, MA | Positive - PD | Risk |
|  | ^190^ | Blood and urine samples were collected as part of the surveys’ examination components | National Death Index | USA National Health and Nutrition Examination Survey III | Positive - AD | Progression |
|  | ^191^ | Blood samples included as part of National Health and Nutrition Examination Survey dataset | Consortium to Establish a Registry for Alzheimer’s Disease Word List Learning and Digit Symbol Substitution Test | 2011-2012 National Health and Nutritional Examination Survey | Positive | N/A** |
|  | ^192^ | Dental amalgam fillings included in the national health insurance research database (NHIRD) | International Classification of Diseases, Ninth Revision, Clinical Modification (ICD-9-CM) code 331.0 | Longitudinal Health Insurance Database  Taiwan | Positive - AD | Risk |
| PAH | ^206^ | Urinary samples provided urinary biomarkers | Digit symbol substitution test | 2001-2002 National Health and Nutritional Examination Survey | Positive | N/A** |
|  | ^207^ | Urinary concentrations of PAH metabolites | Questionnaire-based psychometric testing, anthropometric and blood pressure measurements, mini-mental state examination (MMSE) | Environmental Pollution-Induced neurological Effects (EPINEF)  Republic of Korea | Positive | Risk |
|  | ^208^ | Morning urine samples and peripheral blood collected | Face-to-face interview by the World Health Organization–recommended Neurobehavioral Core Test questionnaire | Shanxi, China | Positive | N/A** |
|  | ^209^ | High-performance liquid chromatography | The World Health Organization (WHO) Neurobehavioral Core Test Battery (NCTB) | China | Positive | N/A** |
|  | ^210^ | Urine samples collected for VOCs and PAHs metabolite analysis | A symptom questionnaire based on the possible effects of the crude oil composition | Taean, Korea | Not Significant |  |

*Non-significant/significant result determination in the “Significant Association?” section is derived directly from significance criteria set by the authors of the original study. Readers should refer to the original study for more details on statistical significance testing and determine effects of exposure/outcome misclassifications.

** worsened cognitive function; dementia not tested
